# Contrasting features of papillary and chromophobe renal cell carcinoma revealed by whole genome sequencing

**DOI:** 10.1158/1541-7786.MCR-25-0616

**Published:** 2026-05-04

**Authors:** Richard Culliford, Charlie Mills, Daniel Chubb, Ben Kinnersley, Amit Sud, Alex J. Cornish, Lisa Browning, Samuel E. D. Lawrence, Robert Bentham, Anna Frangou, Andreas J. Gruber, Kevin Litchfield, David C. Wedge, James Larkin, Samra Turajlic, Richard S. Houlston

**Affiliations:** 1 Division of Genetics and Epidemiology, The Institute of Cancer Research, London, UK; 2 Department of Cellular Pathology, Oxford University Hospitals NHS Foundation Trust, Oxford, UK; 3 Department of Oncology, University College London Cancer Institute, London, UK; 4 Algebraic Systems Biology, Max Planck Institute of Molecular Cell Biology and Genetics, Dresden, Germany; 5 Algebraic Systems Biology, Centre for Systems Biology Dresden, Dresden, Germany; 6 Department of Biology, University of Konstanz, Konstanz, Germany; 7 The Tumour Immunogenomics and Immunosurveillance Lab, University College London Cancer Institute, London, UK; 8 Manchester Cancer Research Centre, University of Manchester, Manchester, UK; 9 NIHR Manchester Biomedical Research Centre, Manchester, UK; 10 Renal and Skin Units, The Royal Marsden NHS Foundation Trust, London UK; 11 Melanoma and Kidney Cancer Team, The Institute of Cancer Research, London, UK; 12 Cancer Dynamics Laboratory, The Francis Crick Institute, London, UK

## Abstract

The identification of cancer drivers is a cornerstone to delivery of precision oncology. So far sequencing of renal cell cancer (RCC) has largely been confined to the clear cell subtype of RCC. In contrast, sequencing analyses of the less common forms of RCC, papillary RCC (pRCC) and chromophobe RCC (ChRCC), have so far been limited. We analysed whole genome sequencing data on 164 tumour-normal pairs from the Genomics England 100,000 Genomes Project, providing a comprehensive, high-resolution map of copy number alterations, structural variation, and key global genomic features, including mutational signatures, intra-tumour heterogeneity and analysis of extrachromosomal DNA formation. Our research establishes correlations between genomic alterations and histological diversification and the extent to which genetically-mediated immune escape contributes to the development of these RCC subtypes.

**Implications:**

We demonstrate the distinctive genetics which characterises pRCC and ChRCC and how this information has the potential to inform patient treatment and clinical trials.

## Introduction

Most kidney cancers are renal cell carcinomas (RCCs). The World Health Organization (WHO-v5) classification of RCC recognizes more than 20 subtypes (1) with 70-90% having clear cell histology (ccRCC). There has been significant progress in the treatment of ccRCC over the last 20 years (2). In contrast, less progress has been made in the treatment of papillary RCC (pRCC) and chromophobe RCC (ChRCC), which account for 13–20% and 5–7%, of RCC cases respectively (3).

The need to understand cancer biology and aetiology, inform development of novel therapies and better predict patient outcomes, has been a motivation in sequencing studies of cancer. So far these studies of RCC have largely been confined to ccRCC (4–6) and studies of pRCC and ChRCC have generally been limited to exome sequencing small numbers of cases (7). To address this shortcoming we report the analysis of whole genome sequencing (WGS) data generated on tumour-normal pairs, from 164 patients recruited from NHS Genomic Medicine Centres (GMCs) across England, as part of the Genomics England 100,000 Genomes Project (100KGP, RRID: SCR_010502; refs 8,9). Our study provides an extensive analysis of the genomic landscape, mutational processes, and clonal architecture underlying the development of ChRCC and pRCC.

## Materials and Methods

### Patients and ethics

The cohort (100kGP, release v14) comprised tumour-normal (T/N) sample pairs with primary ChRCC and pRCC recruited through 13 GMCs. The study was conducted as part of the 100kGP, approved by the East of England-Cambridge South Research Ethics Committee (reference:14/EE/1112) with all patients providing written informed consent. Patients were routine surgical cases and tumour pathology was reported by diagnostic histopathologists at contributing centres. Histology of tumours was initially as per WHO-v4 (10) but herein was updated to WHO-v5 (1). DNA, extracted from EDTA-venous blood samples, served as a source of the patient germline. To avoid sequencing bias associated with polymerase-chain-reaction (PCR) amplification we restricted our analysis to WGS data generated from PCR-free, fresh-frozen tumours from 103 pRCC patients (67 male; median age 62, range 37-87) and 61 ChRCC patients (24 male; median age 62, range 26-83) (Supplementary Table S1-2). We subdivided ChRCC cases into “eosinophilic” (n=13) and “classical” (n=48) (Supplementary Table S2). Cognisant of the emerging molecular subtypes of RCC we sought evidence of *TSC1/TSC2* mutations in eosinophilic cases or SDH alterations, which would justify reclassification as eosinophilic solid and cystic RCC or SDH-deficient RCC respectively. No TSC mutations were detected in any of the eosinophilic cases and while one ChRCC was shown to have a *SDHA* somatic mutation, this was predicted to be benign (Supplementary Methods), thereby precluding tumour reclassification (11).

### Whole genome sequencing

Illumina Inc. conducted 150 base pair (bp) paired-end WGS using HiSeq X technology. Germline and tumor samples were sequenced to an average depth of 30x and 100x, respectively. Tumour purity and ploidy were estimated as per Van Loo *et al*. (12), as part of a pipeline incorporating Battenberg (RRID:SCR_017098, v2.2.8; ref 13) for copy number alteration calling (CNA) and whole genome duplication (WGD) in tumour samples (Supplementary Methods). The median tumour purity was 0.79 (range 0.21-0.97) and 0.74 (0.20-0.98) for ChRCC and pRCC respectively (Supplementary Table S2). Further details on sample curation, tumour purity, and WGS are provided in Supplementary Methods.

### Statistical analysis

We used IntOGen (RRID: SCR_027727; ref 14) to identify coding drivers (Supplementary Methods). We searched for non-coding drivers in core promoters, distal promoters, UTRs and splice regions using OncodriveFML (RRID: SCR_027731; ref 15) and ActiveDriverWGS (RRID: SCR_027737; ref 16). Positive or negative selection of mitochondria (mt) mutations in 12 mt protein-coding genes (excluding *MT-ND6* due to strand bias) was inferred using dNdScv (RRID: SCR_017093; ref 17). Pathways containing driver genes were referenced to PubMed and interrogated using ActivePathways (RRID: SCR_027736; ref 18) and MSigDB (RRID: SCR_016863, v7.5.1; ref 19). The evolutionary timings of driver mutations were estimated using MutationTimeR (RRID: SCR_027739, v0.99.3; ref 20) and ordered using a league model approach, whereby multinomial distributions describing the expected ordering of pairs of driver mutations are repeatedly sampled to generate the ordering of mutations (Supplementary Methods).

Recurrent arm-level CN events, focal amplifications and focal deletions, were identified using GISTIC (RRID: SCR_000151, v2.0.2.3; ref 21) (Supplementary Fig. S1-2, Supplementary Table S3). Structural variants (SVs) were identified from a consensus of three SV calling algorithms, and classified as simple or complex using ClusterSV (RRID: SCR_027722; ref 22) with hotspots assigned as per Glodzik *et. al*. (23) (Supplementary Fig. S3, Supplementary Methods). Amplicon structures were identified using AmpliconArchitect (RRID: SCR_023150; ref 24). Telomere length of germline and tumour DNA were estimated from their respective bam files using TelomereCat (RRID: SCR_027747, v3.3.0; ref 25), adopting default parameters.

*De novo* extraction of single-base-substitution (SBS) and insertion and deletion (ID) mutational signatures, including decomposition to known COSMIC signatures (RRID: SCR_002260, v3.2; ref 26), was performed using SigProfilerExtractor (RRID: SCR_023121, v1.1.4; ref 27). We used mSINGS (RRID: SCR_027728; ref 28) and HRDetect (RRID: SCR_027726; ref 29) to identify mismatch repair deficient (dMMR) and homologous recombination deficient (dHR) tumours.

Genomes were HLA-typed using POLYSOLVER (RRID: SCR_022278; ref 30) and neoantigens predicted using pVAC-Seq (RRID: SCR_025435, v3.1.2; ref 31). We considered tumours as having genetically predicted immune escape on the basis of a non-synonymous mutation, or loss-of-heterozygosity (LOH) in any HLA Class-I gene, or an inactivating mutation in one of 22 antigen presenting genes (APGs). T-cell ExTRECT (RRID: SCR_027742; ref 32) was used to estimate the T-cell receptor α-chain (TCRA) T-cell fraction within tumour samples from WGS, which was used as a proxy measurement of T-cell infiltration. Further details of bioinformatic analyses are provided in Supplementary Methods.

To compare frequencies of categorical variables with molecular features we used Fisher’s exact test or logistic regression. For quantitative traits we used t-tests, Wilcoxon rank sum test, linear regression or negative binomial regression. In all cases we considered a two-sided *P*-value <0.05 as being statistically significant.

## Data and code availability

The data analyzed in this study are available from Genomics England Ltd. Restrictions apply to the availability of these data, which were used under license for this study. The data was deposited in the National Genomic Research Library and can be accessed via the Genomics England Research Environment secure cloud workspace. Access can be obtained by first applying to become a member of either the Genomics England Research Network (https://www.genomicsengland.co.uk/research/academic) or the Discovery Forum (industry partners https://www.genomicsengland.co.uk/research/research-environment). The process for joining the network is described at https://www.genomicsengland.co.uk/research/academic/join-gecip and consists of the following steps:

1. Your institution will need to sign a participation agreement available at https://files.genomicsengland.co.uk/documents/Genomics-England-GeCIP-Participation-Agreement-v2.0.pdf and email the signed version to gecip-help@genomicsengland.co.uk.
2. Once you have confirmed your institution is registered and have found a domain of interest, you can apply through the online form at https://www.genomicsengland.co.uk/research/academic/join-gecip where you can specify the reason for access and expected timeframe that you wish to have access. Once your Research Portal account is created you will be able to login and track your application.
3. Your application will be reviewed within 10 working days.
4. Your institution will validate your affiliation.
5. You will need to complete the online Information Governance training and will be granted access to the Research Environment within 2 days of passing the online training.

The processed clinical and genomic data applied to the investigation are available in the Research Environment within the folder /re_gecip/shared_allGeCIPs/rculliford/RCC_landscape. At present, there is no proposed end date for data access within the research environment. All other public/private datasets used in the study, including corresponding download links and version numbers, can be found in Supplementary Table S4.

After completion of the instructions given by the Data Availability statement, code to allow for reproducibility of results and figures are available in the research environment within the folder /re_gecip/shared_allGeCIPs/rculliford/RCC_landscape. Sources of each software package and externally downloaded data are detailed in Supplementary Table S4.

## Results

None of the patients were carriers of a pRCC (*i*.*e. MET, PTEN, FH*) or ChRCC susceptibility gene (*i*.*e. TSC1, TSC2, PTEN* or *FLCN*; Supplementary Table S5) (33). As previously documented (7), ChRCCs displayed a low tumour mutational burden (TMB) (median 0.61/Mb, median ploidy 1.7, and weighted genome instability index (WGII) 34.8%). In contrast, pRCC was characterised by a higher TMB (1.79/Mb, ploidy 2.19, WGII of 22.6%), similar to ccRCC (6). Germline samples tended to have a longer telomere compared to their matched tumour in both the ChRCC (mean germline TL= 4700, mean tumour TL = 4151, t-test *P*=0.02) and pRCC (mean germline TL = 4567, mean tumour TL = 3542, t-test *P*=2.19 × 10^−14^) cohorts as expected. There was no significant difference in the mean germline-tumour telomere difference between eosinophilic and classical ChRCC (eosinophilic ChRCC mean telomere difference= -700, classical ChRCC mean telomere difference = -509, t-test *P*=0.76) (Supplementary Fig. S4, Supplementary Table S2).

### Structural alterations

**Figure 1:**
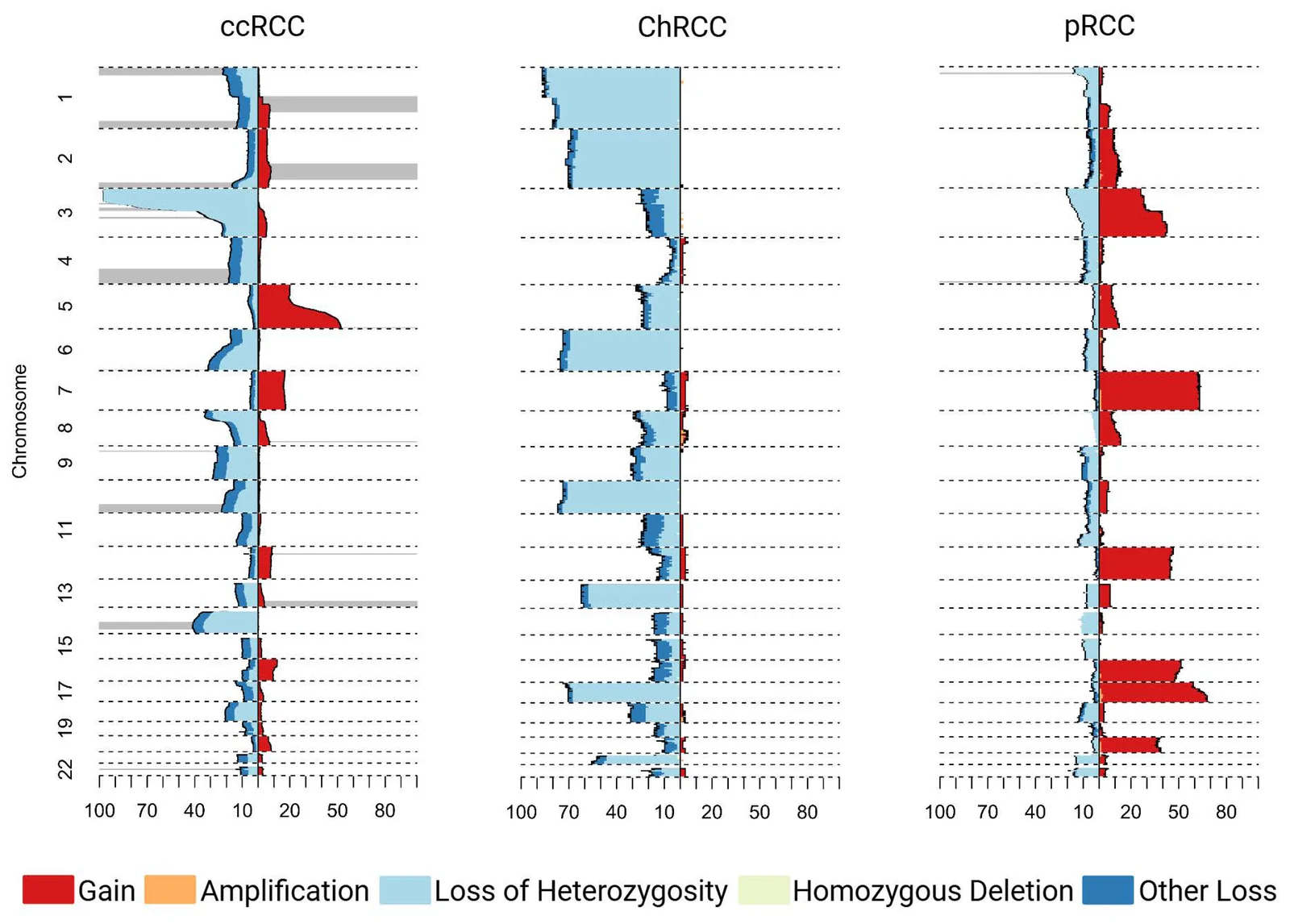
Contrasting copy number profiles of ccRCC, ChRCC and pRCC. Information on ccRCC copy number from Culliford *et al*. (2024).

ChRCC and pRCC tumours showed distinctive chromosomal profiles - markedly different from ccRCC (Fig. 1, Supplementary Table S6-7) (4,6). As previously reported (7,11,34), most classical ChRCCs showed loss of chromosomes 1, 2, 6, 10, 13, 17 and 21, in contrast to the eosinophilic cases (Supplementary Fig. S5a-b, S6a-b). In comparison, pRCCs showed gain of chromosomes 7 and or 17 (76%, 78/103), accompanied by gain of chromosomes 3, 12, 16, and 20, albeit at lower frequency (Fig. 1, Supplementary Table S6).

WGD was identified in 20% (12/61) of ChRCC tumours, similar to ccRCC (6), but only 5% (5/103) of pRCC. Eosinophilic ChRCC tumours were also likely to have WGD detected in comparison to their non-eosinophilic counterparts (1/13 vs 11/48). In contrast to many other cancer types, such as breast and colorectal (35), WGD in ChRCC was not associated with *TP53* mutations (Fisher’s exact test, *P*=1.0) with only 21% (3/14) of *TP53*-mutated tumours also being positive for WGD (Supplementary Table S2).

We identified three SV hotspot regions in ChRCC tumours (Supplementary Table S8, Supplementary Fig. S8a-c); one defined by chr5:12448-1417975, which encompassed *TERT* and *SDHA*, in five tumours. None harboured a coding change in *TERT*. However, a missense mutation in *SDHA* was detected in one cancer. No SV hotspot regions were identified in pRCC tumours. ecDNA has been associated with oncogene amplification and poor outcome in some cancers. Complex rearrangements were identified in 5% (3/61) of ChRCC and 16% (17/103) of pRCC, implicating *CDK6*. However, in contrast to many cancers (36), ecDNA was not a common feature of either RCC subtype only being identified in 3% (2/61) of ChRCC and 7% (7/103) of pRCC (Supplementary Fig. S9).

### Cancer-drivers

We identified seven genes significantly mutated in ChRCC, (*BAP1, CDKN1A, CSPG4, MAP3K13, MTOR, TP53*, and *WNK2*), and 14 genes in pRCC (*ARID1A, CACNA1D, ELF3, KDM5A, KIAA1549, KMT2C, LEPROTL1, MAP3K1, MET, MTOR, NFIB, PBRM1, SETD2, UBR5*). None of these drivers were associated with recurrent hotspot mutations and mutational frequencies were broadly concordant with published data (Supplementary Fig. S10a-b, Supplementary Table S9-10). In our analysis there was no evidence for mutual exclusively in any of these driver genes (Fisher exact text, *P*<0.05).

**Figure 2:**
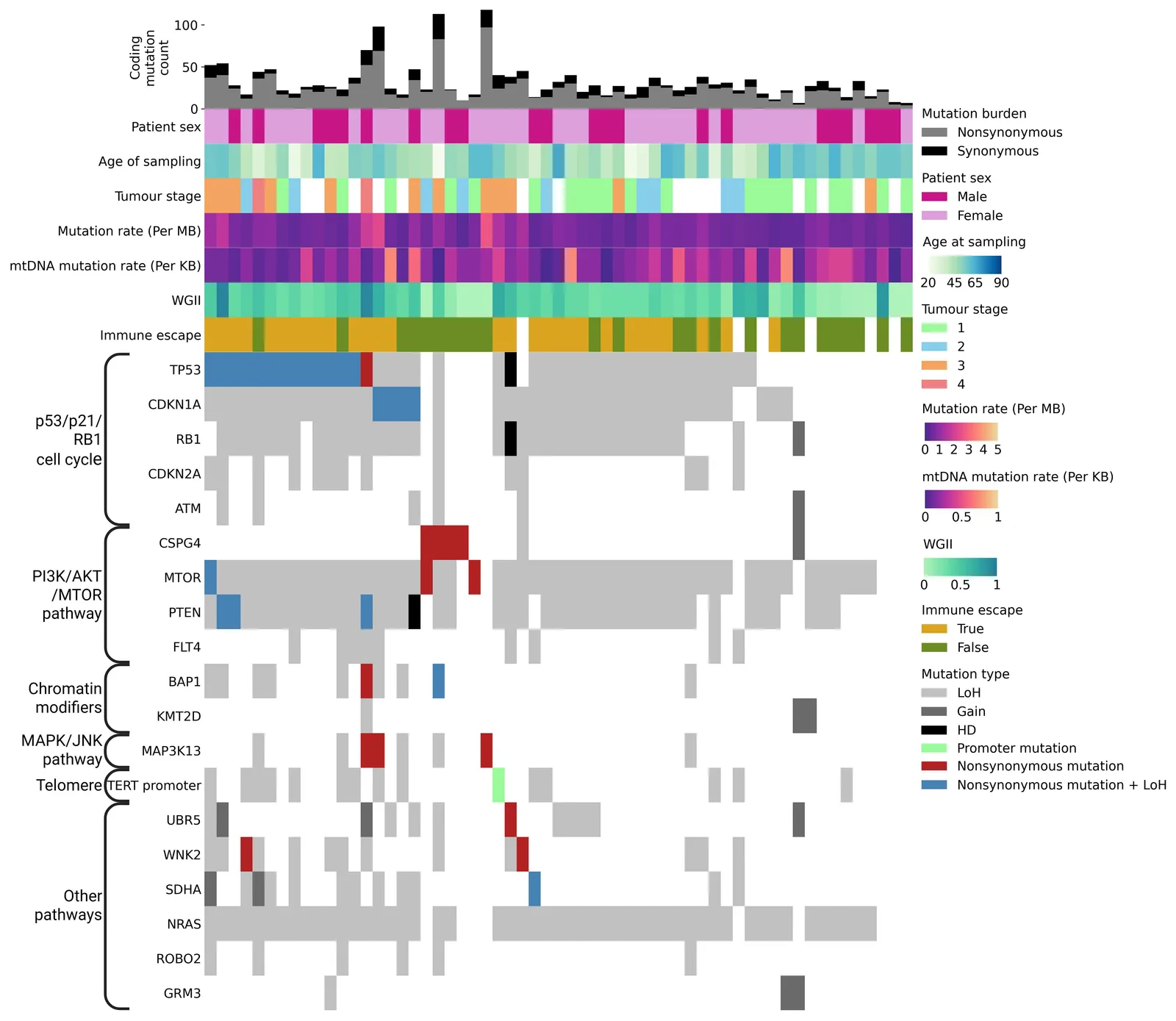
Relationship between genomic profile and clinical features for ChRCC (n=59). Samples ordered by highest coding mutational burden for each histology. Genes are ordered by major pathways, followed by mutation status and then copy number alteration. LoH: Loss of heterozygosity; HD: Homozygous deletion; MB: Megabase; KB: Kilobase; WGII: Weighted genome instability index; mtDNA: mitochondria DNA.

**Figure 3:**
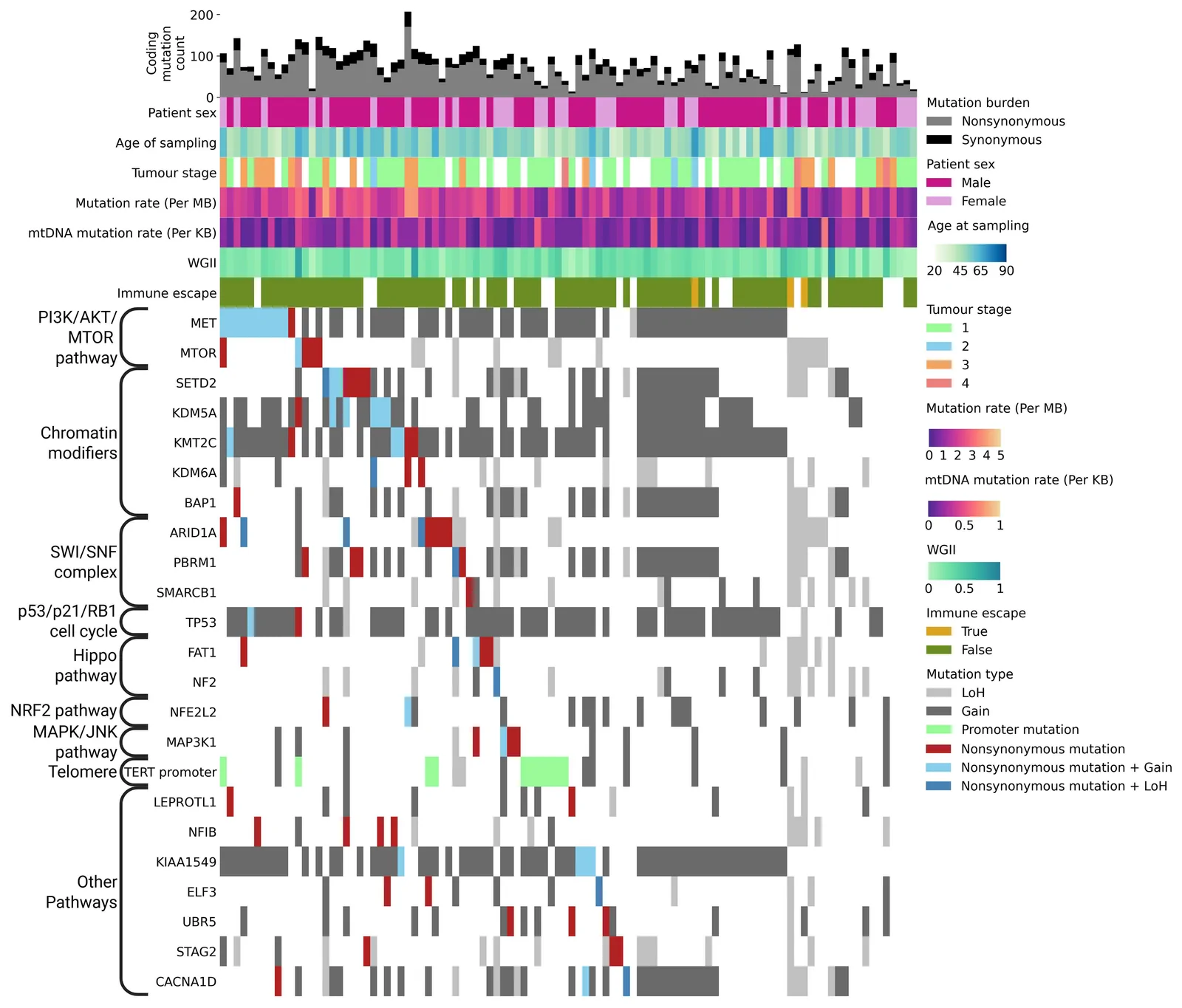
Relationship between genomic profile and clinical features for pRCC (n=102). Samples ordered by highest coding mutational burden for each histology. Genes are ordered by major pathways, followed by mutation status and then copy number alteration. LoH: Loss of heterozygosity; MB: Megabase; KB: Kilobase; WGII: Weighted genome instability index; mtDNA: mitochondria DNA.

We conducted a further search under a restricted hypothesis by considering genes previously implicated in RCC (14,37,38). This analysis provided additional support for the role of 12 genes in ChRCC (*ATM, CDKN2A, FLT4, GRM3, KMT2D, NRAS, PTEN, RB1, SDHA, TSC1, TSC2, UBR5*) and eight genes in pRCC (*BAP1, FAT1, KDM6A, NF2, NFE2L2, SMARCB1, STAG2, TP53*) (Fig. 2-3, Supplementary Table S10). Extending our analysis to consider genes implicated by virtue of focal genomic alterations provided additional support for *ARID1A* in pRCC (Supplementary Table S7).

Considering large-scale chromosomal alterations provided support for reported drivers in ChRCC and pRCC (Supplementary Fig. S11-12, Supplementary Table S6, S9-10). *CDKN1A* (del6p21.2), *PTEN* (del10q23.31), *RB1* (del13q4.2) and *TP53* (del17p13.1) are supported as drivers in ChRCC. In contrast to previous reports (37), our analysis did not provide evidence to implicate *TSC1* and *TSC2* in either classical or eosinophilic ChRCC. In pRCC, alterations implicated *KDM5A* (amp12p13.33), *KIAA1549* (amp7q34) and *MET* (amp7q31.2). In our analysis there was no evidence to support any driver gene in ecDNA.

Searching for non-coding drivers identified the core promoter region of *TERT* as recurrently mutated in 11 pRCC tumours; 10 of which harboured the rs1242535815 mutation, which has been documented to be disease-causing in RCC and other tumour types (39–41) (Supplementary Table S11). The difference in TL between tumour and germline was significantly larger in tumours harbouring *TERT*-promoter mutations (tumour-germline TL difference: -1700 for mutated and -945 wild-type, t-test *P*=0.0068). Presumptive pathogenic mutations identified the *CCDC107* distal promoter region as a driver in pRCC.

**Figure 4:**
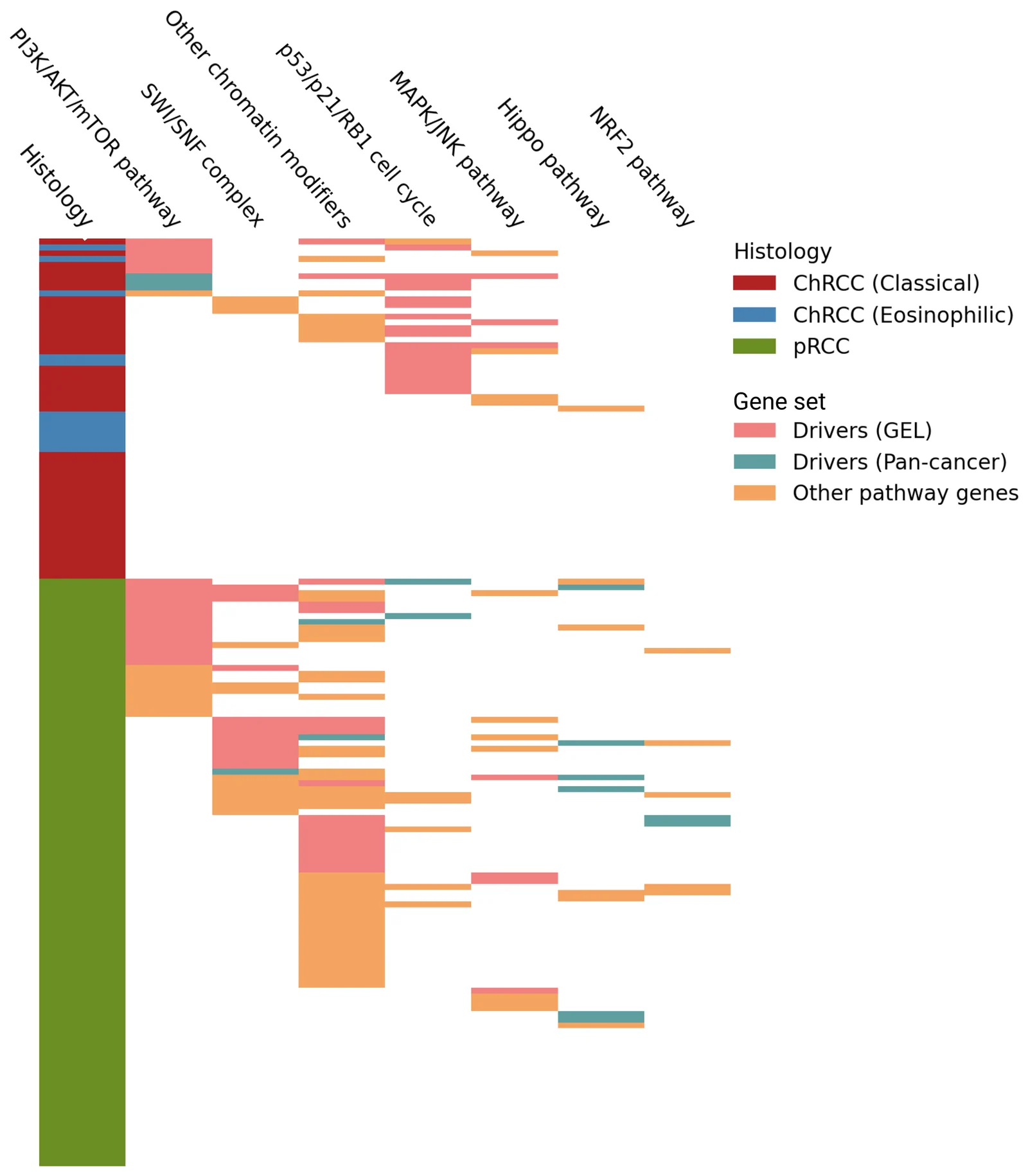
Major biological pathways disrupted in ChRCC (n=59) and pRCC (n=102). Genes in “Pan-cancer” refer to driver genes that are recurrently mutated in Bailey et al. (2018). Genes in “Other Pathway Genes” refer to all other canonical genes, including non-cancer driver genes, within the corresponding cellular pathway (Supplementary Methods). GEL: Genomics England 100,000 Genomes Project.

Our analysis emphasises the differences in gene pathways between the RCC subtypes (42). Specifically, the importance of mutations in the PI3K/AKT/mTOR and p53/p21/RB1-pathway in ChRCC (Fig. 4, Supplementary Fig. S13). There was limited evidence to support mutation of chromatin modifier genes in the development of ChRCC in contrast to pRCC or ccRCC (6). Mutation of *MET* was essentially confined to pRCC. In terms of clonal architecture *MTOR* and *TP53*-related genes were early mutational events in the development of ChRCC. Similarly, mutation of *PBRM1* was an early event in the pRCC tumours. In contrast *ELF3* mutations in pRCC tumours tended to be a late event (Supplementary Fig. S14).

### mtDNA analysis

There is increasing evidence for mt-dysregulation in cancer (43). While mtDNA mutation rates were not significantly different between ChRCC (including eosinophilic) and pRCC, the frequency of mtCN was significantly higher in pRCC tumours (t-test, *P*<0.001; Supplementary Fig. S15a-b, Supplementary Table S2 and S12).

Across histologies we identified mutated genes in complexes I and III under positive selection and genes in complex-IV which were typically negatively selected. Irrespective of heteroplasmy, *MT-CYB* showed evidence of being under positive selection for missense mutations in ChRCC (dN/dS=1.84, *P*=0.03). *MT-ND2* showed a higher proportion of high VAF missense mutations in pRCC (dN/dS=4.91, *P*=0.0048). While *MT-ND4* was positively selected in ChRCC for low VAF missense mutations (dN/dS=1.94, *P*=0.02), there was no evidence that ChRCC tumours were more likely to be *MT*-*ND4*-positive (Fisher’s exact test *P*=1) or *MT-ND5*-positive (Fisher’s exact test *P*=1.0) than pRCC tumours (Supplementary Table S13-14).

### Mutational signatures

**Figure 5:**
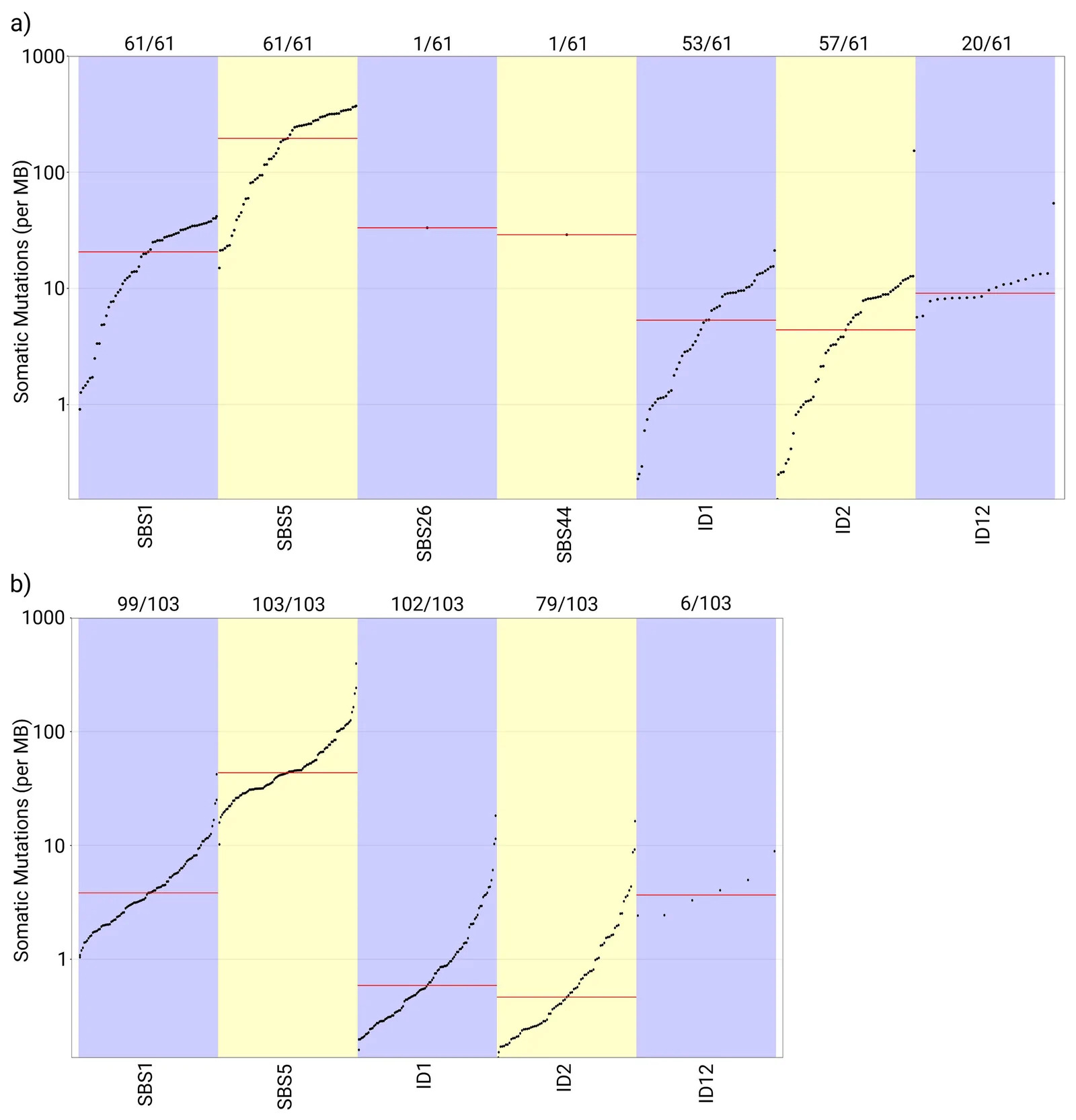
Mutation rate associated with each COSMIC signature. (a) ChRCC (n=61), and (b) pRCC (n=103). SBS1: Spontaneous deamination of 5-methylcytosine (clock-like signature); SBS5: Unknown (clock-like signature); SBS26: Defective DNA mismatch repair; SBS44: Defective DNA mismatch repair; ID1: Slippage during DNA replication of the replicated DNA strand; ID2: Slippage during DNA replication of the replicated DNA strand; ID12: Unknown aetiology; MB: Megabase.

To gain insight into mutational processes, we extracted decomposed-COSMIC SBS and ID mutational signatures (RRID:SCR_002260, v3.2) for each tumour (Fig. 5a-b, Supplementary Fig. S15, Supplementary Table S15). In both tumour types, SBS1 and SBS5, resulting from clock-like mutagenic processes, were ubiquitous. One hypermutated ChRCC tumour with the *POLE* and *POLD1* mutations (Supplementary Table S5, Supplementary Fig. S13) displayed SBS26 and SBS44, reflective of dMMR. ID1 and ID2, a consequence of replication slippage, was frequent in ChRCC, with a lower presence of ID12 (unknown aetiology). The same ID signatures were also a feature of pRCC, albeit ID1 was at a higher frequency compared to ChRCC (99% vs. 86.9%) while ID2 (76.7% vs. 93.4%) and ID12 (5.8% vs. 32.8%) showed lower frequency (Fig. 5a-b, Supplementary Fig. S14, Supplementary Table S15). None of the tumours showed evidence of dHR.

### Immune evasion

We predicted 2,051 class-I neoantigens across 55 ChRCC (n=867, 0-607/tumour, median 1) and 86 pRCC (n=1184, 0-65/tumour, median 9) tumours; >95% a consequence of frameshift variants. There was no difference in the distribution of neoantigen counts between eosinophilic and classical ChRCC tumours (Wilcoxon Rank Sum *P*=0.42). We detected LOH of HLA in 48% (n=29) of ChRCC and 4% (n=3) of pRCC tumours. There was no association between indel burden and HLA allele status in ChRCC or pRCC and no tumours had HLA mutations. Only one ChRCC harboured an APG mutation while no pRCC tumours had APG mutations. Collectively these data showed 49% of ChRCC had evidence of genetically predicted immune evasion (albeit primarily a consequence of chromosome 6 loss), but was only a feature of 4% of pRCCs. There was a positive correlation between mutation of *TP53* and genetically predicted immune escape (OR = 7.4, Fisher’s exact test, *P* = 0.01) in ChRCC.

### Clinico-pathological relationships

In both ChRCC and pRCC, age at sampling was correlated with TMB (univariate linear regression *P*=0.04) and SV burden (univariate negative binomial regression *P*=0.01), while stage was positively associated with chromosomal aberrations (Fisher’s exact test *P*=0.01). SV burden and T cell infiltration was positively associated with higher grade in pRCC (univariate linear regression *P*=4.0 × 10^−12^ and Fisher’s exact test *P*=0.04 respectively). Furthermore, higher stage ChRCC and pRCC tumours were more likely to have *TP53* (Fisher’s exact test *P*=0.02) and *MET* mutations (Fisher’s exact test *P*=0.014) respectively.

**Figure 6:**
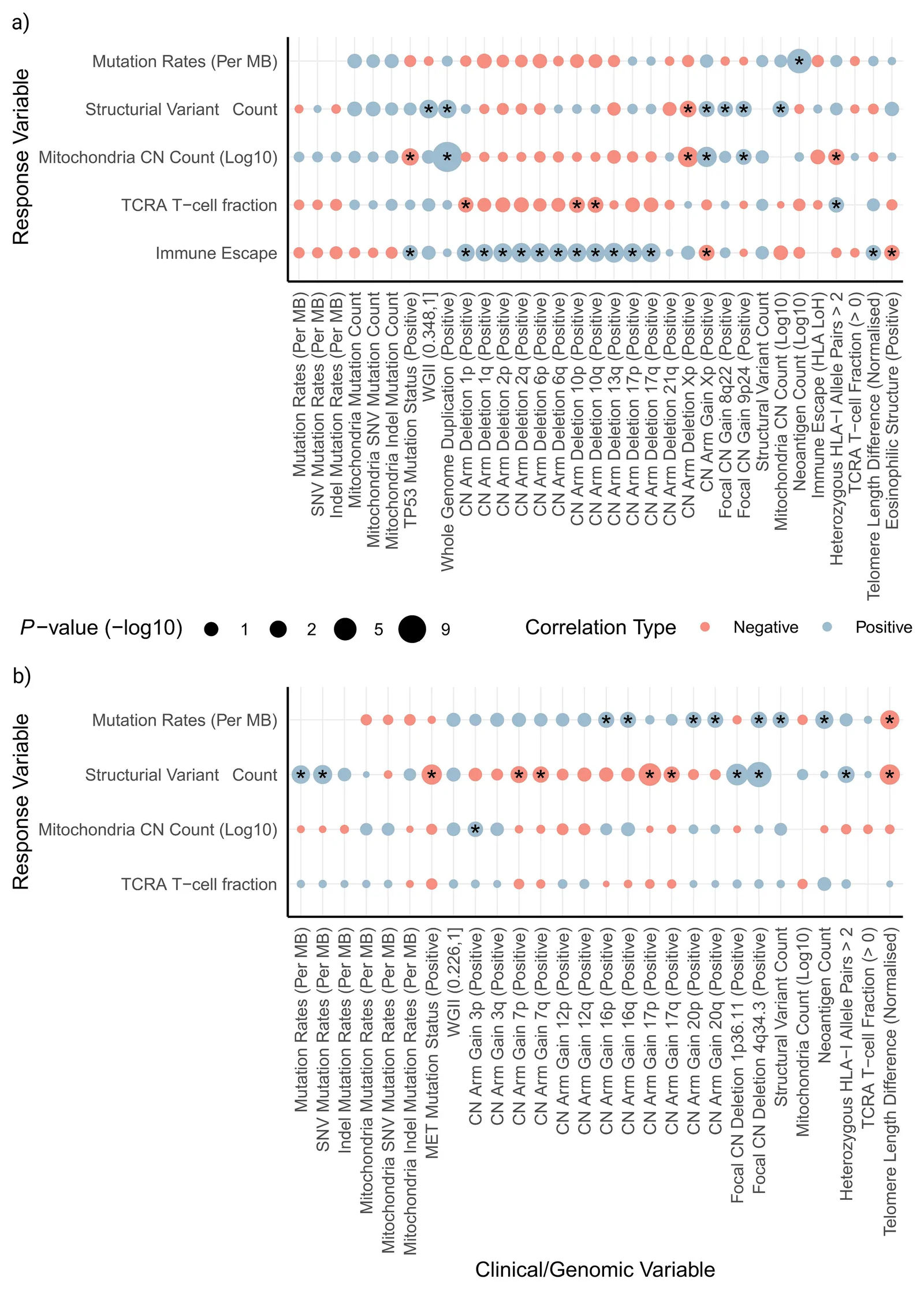
Summary of clinical correlations adjusted for sex, age of sampling and stage. a) chromophobe RCC (n=61) and b) papillary RCC (n=103). SNV: Single nucleotide variant; CN: Copy number; MB: Megabase, WGII: Whole genome instability index; TCRA: T-cell receptor-alpha; *: *P* < 0.05.

Increased mtCN was associated with WGD in ChRCC tumours (multivariate linear regression, *P*=6.49 × 10^−12^). Across all tumours, T-cell infiltration was not associated with neoantigen burden, but increased number of heterozygous HLA-I allele pairs was associated with increased infiltration in ChRCC (multivariate logistic regression *P*=0.04). ChRCC tumours which showed evidence of LoH-induced immune escape were more likely to be *TP53*-mutated (multivariate logistic regression *P*=0.04), a feature irrespective of neoantigen burden (Fig. 6a-b, Supplementary Tables S16-20).

### Clinical actionability of genomic features

We assessed the actionability of driver genes by referencing the OncoKB Knowledge Base(RRID: SCR_014782, v3.11; ref 44). For ChRCC and pRCC we identified 1 and 2 unique Onco-KB annotated alterations respectively in *MTOR* that were targetable (Level 3a-4). This included L2427Q identified in one ChRCC and pRCC, which has clinical evidence (Level 3b) in RCC of being targetable by temsirolimus. Standard of care treatment of amplified *MET*-positive non-small lung cancer suggests 63% (65/103) of *MET*-amplified positive pRCC might be eligible for capmatinib, crizotinib or tenpotinib. In pRCC, there was Level 4 evidence that inactivating mutations in *ARID1A* could be targetable with PLX2853 or Tazemetostat. To complement this analysis we first examined the COSMIC Mutation Actionability in Precision Oncology database (RRID:SCR_002260; ref 26) highlighting an additional 30 unique mutations as potentially targetable. Notably there are ongoing early phase trials targeting *MET* in pRCC, based on Cabozantinib and Savolitinib, and PF-04217903 targeting M1250T. Additionally, solid tumours with *ARID1A, BAP1* and *MTOR* mutations are the subject of ongoing trials (Supplementary Table S21). To broaden our search for novel therapeutic avenues we examined whether any of the RCC driver genes might be candidates for synthetic lethality using SYLVER (RRID:SCR_027764, ref 45), identifying that *PBRM1* deficiency may be amenable to receptor tyrosine kinase inhibition.

## Discussion

We have sought to advance our understanding of non-ccRCC subtypes by analysing WGS data from ChRCC and pRCC tumours. Our analysis emphases the very distinctive genomic profile of these RCC tumour subtypes. As well as confirming established drivers we further highlight p53/p21/RB1 (G1/S) cell cycle, PI3K/AKT/mTOR and chromatin modifier pathways as being central to the biology of ChRCC. In comparison, *MET* and chromatin modifiers were a central feature of pRCC. Furthermore, our analysis validates previous reports of structural abnormalities associated with these non-ccRCC tumours. We did not confirm the reported very high rate of WGD in ChRCC of 60%, however this previous work was based on an analysis of only 10 tumours (46). In contrast to our ccRCC signature analysis (6), this study did not provide support to implicate either tobacco smoking or exposure to aristolochic acid as a risk factor for either ChRCC or pRCC.

Our observations reinforce the desirability of subtype-specific treatment paradigms for non-ccRCC. To investigate the prospect of targeting specific driver mutations we queried OncoKB, which is regularly curated by an expert panel and therefore generally considered to reflect the current state of knowledge. Since other investigators have reported a higher targetable variant detection rate by applying multiple tools to annotate variants, we also made use of the COSMIC resource. Most of the alterations we describe as being actionable are based on clinical evidence from other cancers or biological plausibility. As per previous reports, most of the targetable alterations we identified are within PI3K/mTOR pathway genes. While the mTOR inhibitors temsirolimus and everolimus have received regulatory agency approval, their clinical benefit in non-ccRCC tumours is modest (47).

Whereas the management of ccRCC has been transformed by immune checkpoint inhibitors (ICIs), a recent review of ICI clinical trials has concluded only 6% of ChRCC respond to ICIs (48). While one of the ChRCC cases had a hypermutated phenotype and thus might be amenable to ICI, in our analysis most ChRCCs were immunologically cold, showing a low mutational rate, high HLA loss and limited T-cell infiltration. Moreover, our analysis of ChRCC aligns with previous reports of *TP53-*mediated immune evasion, as a general feature of cancer (49). Recent data from Phase-II trials studies suggests that immune checkpoint inhibitors such as ipilimumab/nivolumab may hold promise as first line treatment for patients with metastatic non-ccRCC cancers (50,51). Notably in the case of pRCC the lack of genetically predicted immune escape may underpin the observation that patients treated with ICI have been reported to have a better outcome than patients solely receiving a tyrosine kinase inhibitor (TKI) as first line therapy (51).

Because of its low incidence the treatment for non-ccRCC has largely been based on evidence from small phase II clinical trials or extrapolated from successful therapies in ccRCC. Nevertheless, several Phase II trials have shown the promise of MET inhibitors in the management of metastatic pRCC, demonstrating superior clinical activity as compared to conventional TKIs and leading to less reliance on solely targeting of the PI3K-axis (Supplementary Table S21). An important caveat to our analysis is that the genetic profiles we derived are of a single region, which has potentially limited our ability to detect clinically important sub clonal targetable alterations.

In many other cancers, a high mutational and neoantigen burden have been linked to better overall survival and responsiveness to checkpoint inhibitors presumably reflecting native immune responsiveness. Conversely, immune escape of tumours by virtue of HLA loss or mutation of APGs has been linked to worse clinical outcome. Given that immune escape was a more common feature of ChRCC this may explain the poorer clinical response of metastatic ChRCC tumours compared to pRCC and other non-ccRCC tumours to PD-1 inhibitors in retrospective analyses or clinical trials (i.e. pembrolizumab in the KEYNOTE 427-b study) (52,53).

In conclusion, our observations reinforce calls that the distinctive genetics associated with subtypes of RCC tumours has potential value in informing the design of clinical trials of RCC (54).

## Supplementary Material

S1 Figure

S1 Table

S10 Table

S11 Table

S12 Table

S13 Table

S2 Figure

S3 Figure

S4 Figure

S5 Figure

S6 Figure

S7 Figure

S8 Figure

S9 Figure

S10 Figure

S11 Figure

S12 Figure

S13 Figure

S14 Figure

S15 Figure

S16 Figure

S2 Table

S3 Table

S4 Table

S5 Table

S6 Table

S7 Table

S8 Table

S9 Table

S14 Table

S15 Table

S16 Table

S17 Table

S18 Table

S19 Table

S20 Table

S21 Table

Supp Methods
