# Supplementary figures and images for: Contrasting features of papillary and chromophobe renal cell carcinoma revealed by whole genome sequencing
Source: Mol Cancer Res. Author manuscript; Available in PMC 2026 Jun 15. (PMC13136883; doi:10.1158/1541-7786.MCR-25-0616)

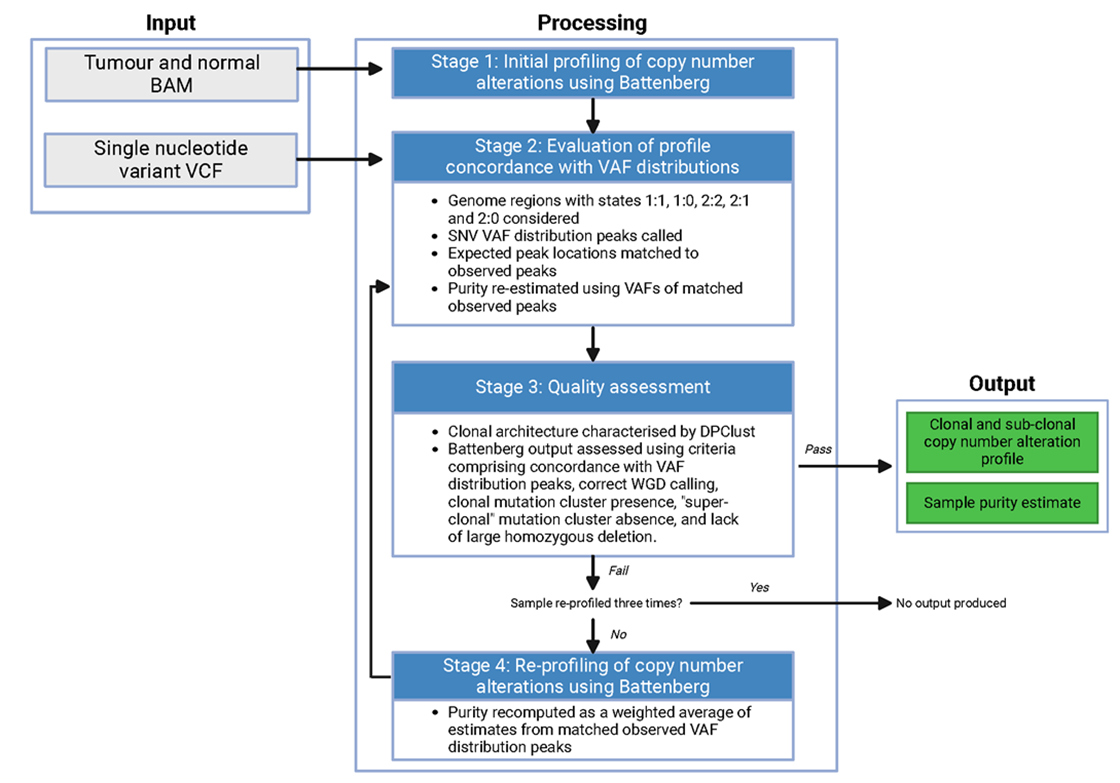

Supplement: S1 Figure [file EMS212285-supplement-S1_Figure.png]

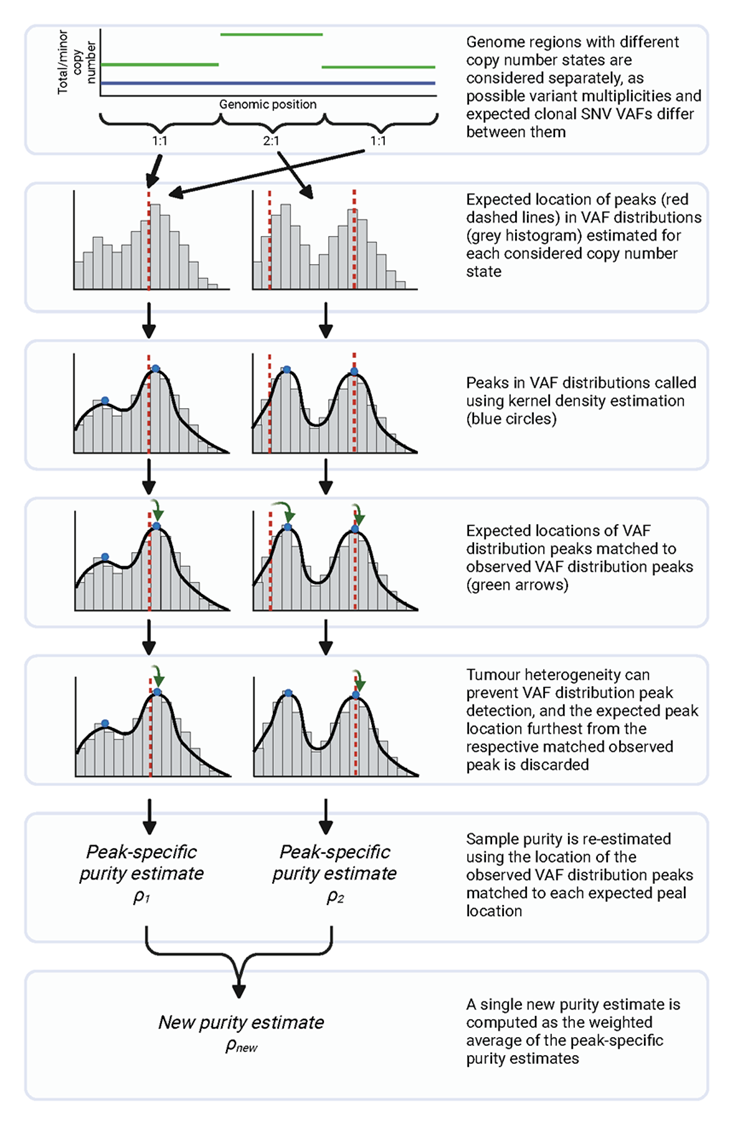

Supplement: S2 Figure [file EMS212285-supplement-S2_Figure.png]

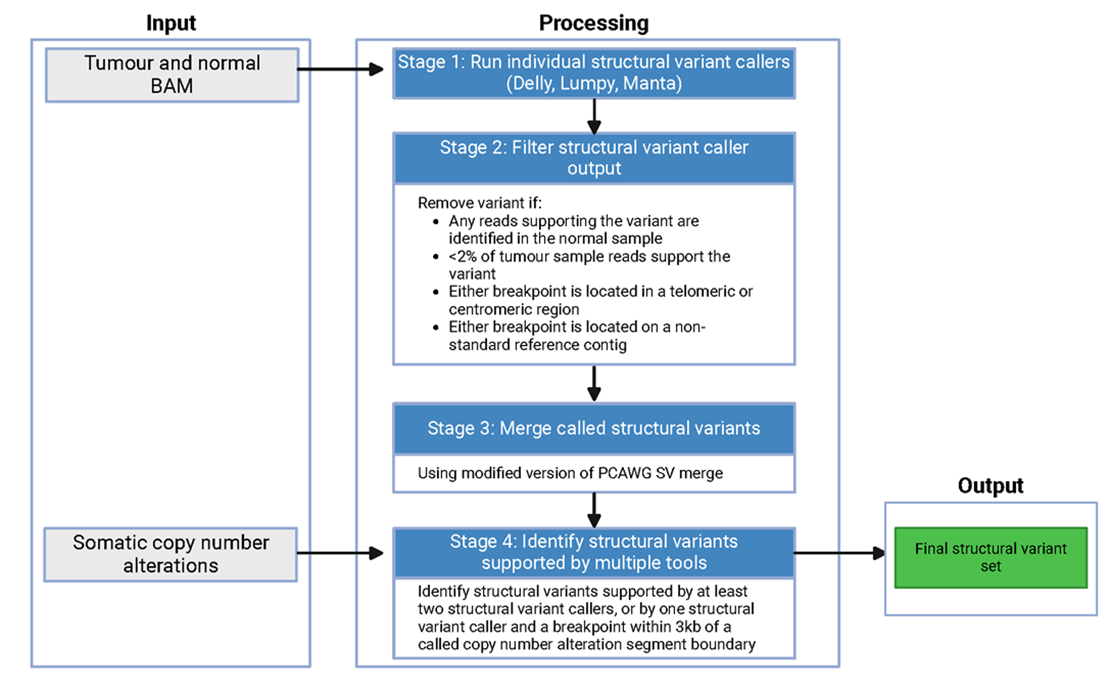

Supplement: S3 Figure [file EMS212285-supplement-S3_Figure.png]

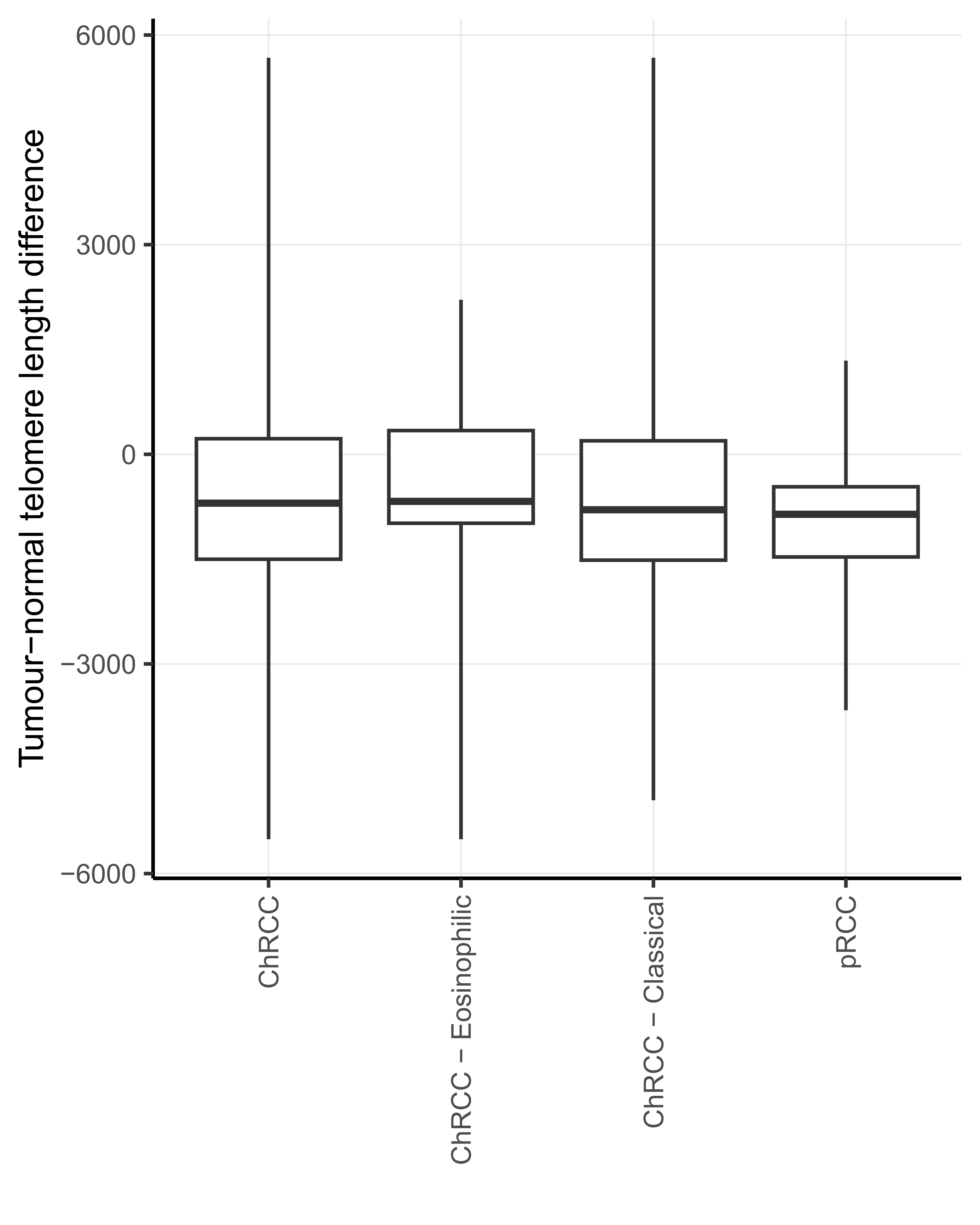

Supplement: S4 Figure [file EMS212285-supplement-S4_Figure.png]

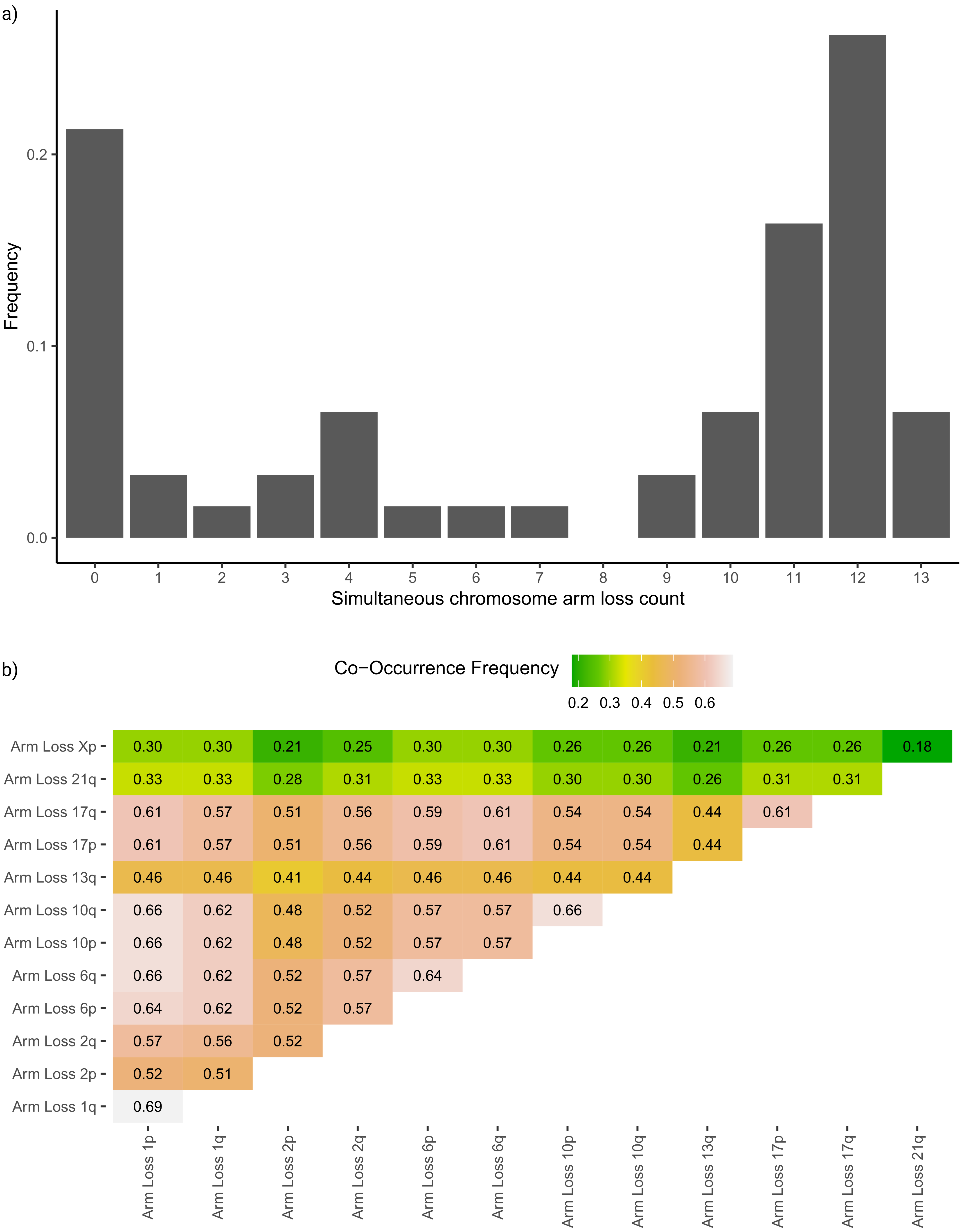

Supplement: S5 Figure [file EMS212285-supplement-S5_Figure.png]

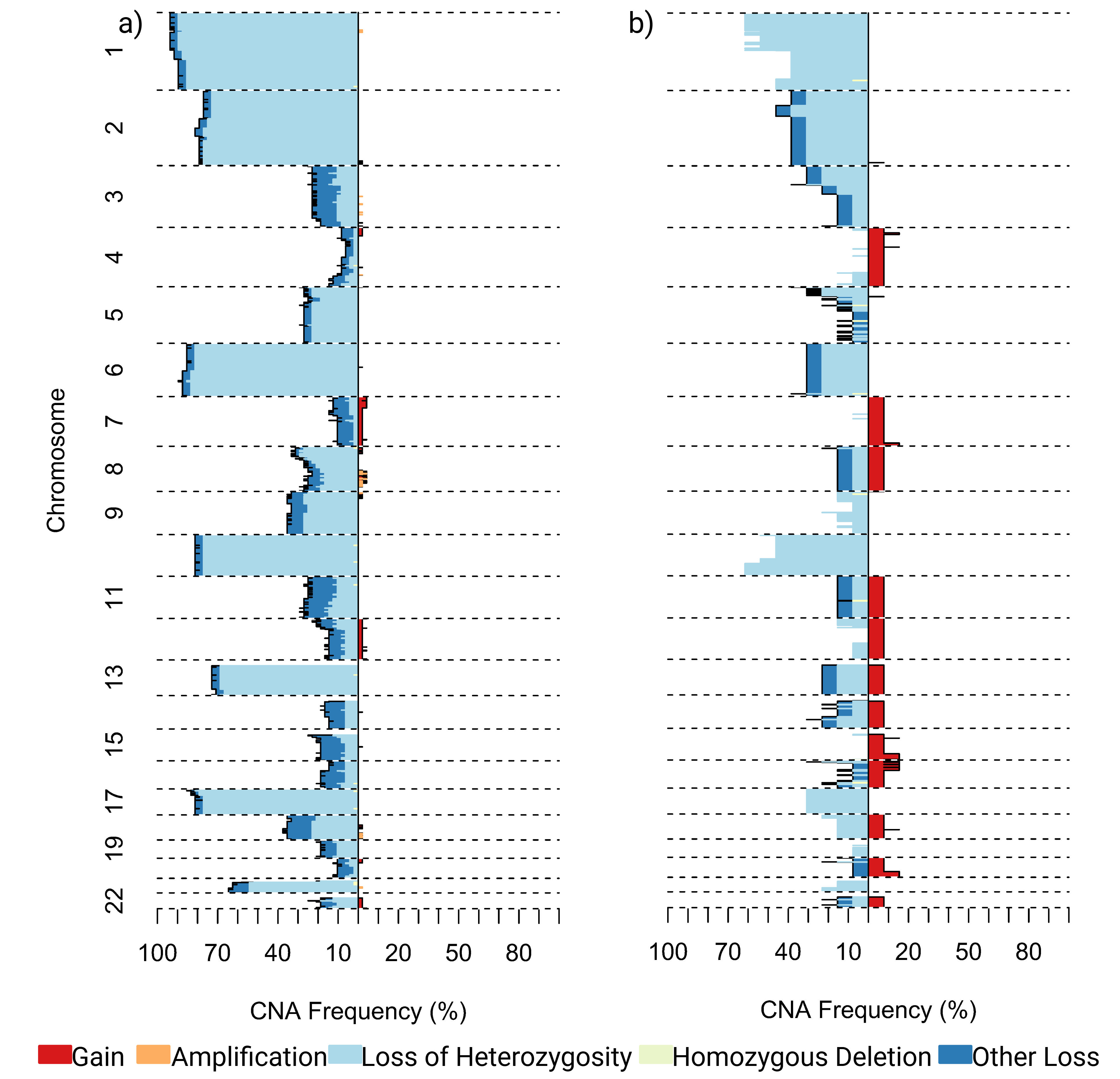

Supplement: S6 Figure [file EMS212285-supplement-S6_Figure.png]

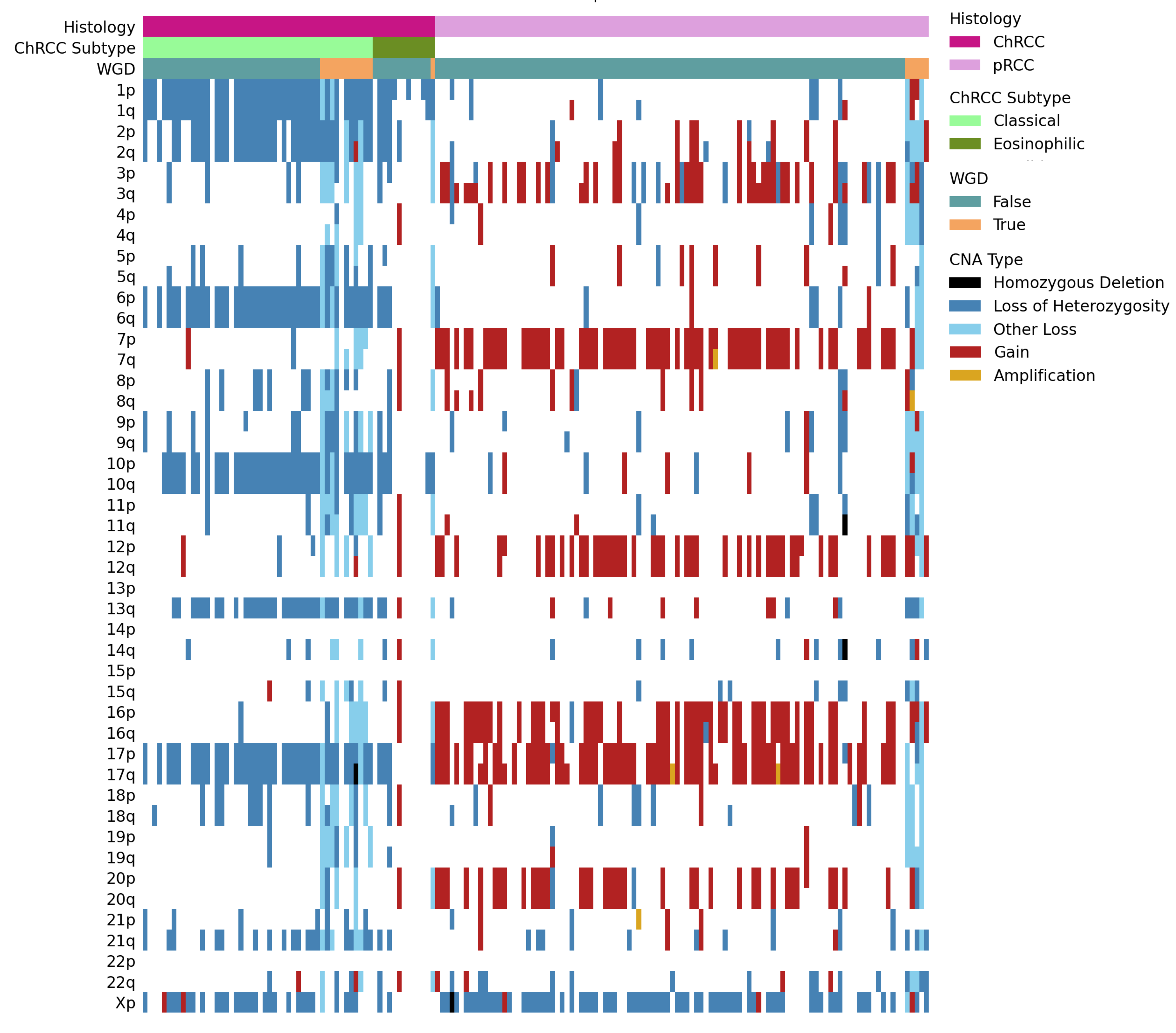

Supplement: S7 Figure [file EMS212285-supplement-S7_Figure.png]

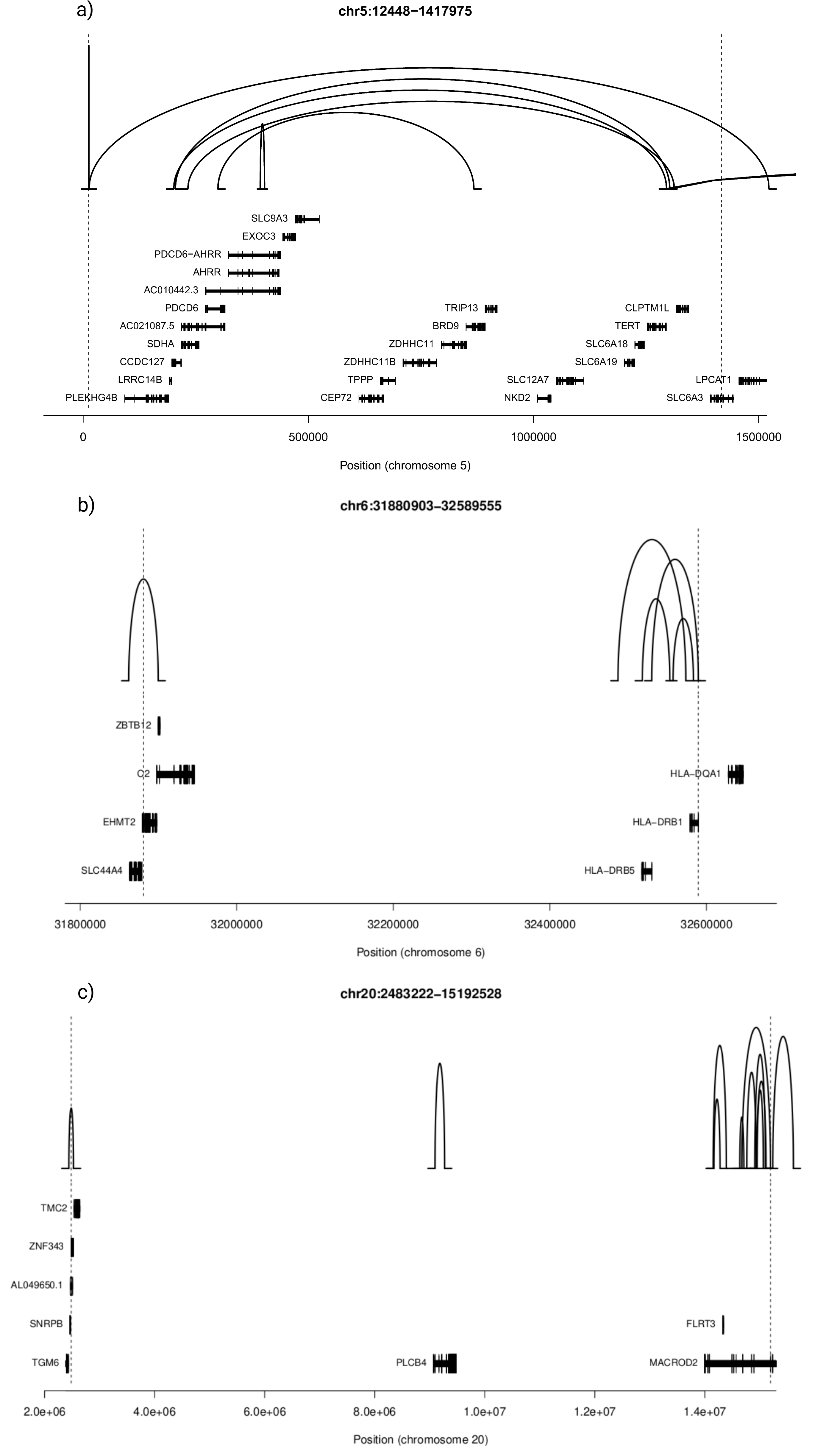

Supplement: S8 Figure [file EMS212285-supplement-S8_Figure.png]

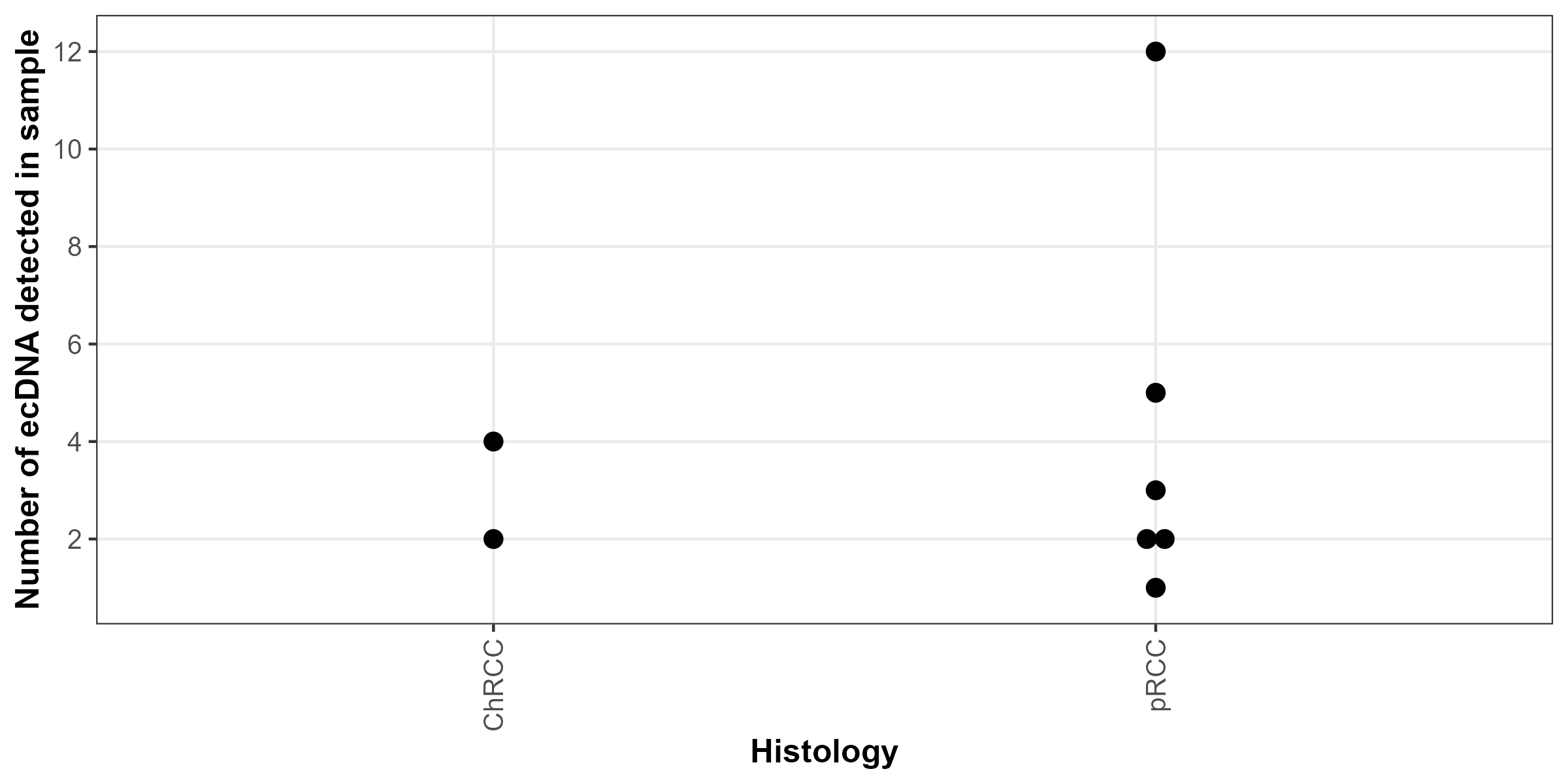

Supplement: S9 Figure [file EMS212285-supplement-S9_Figure.png]

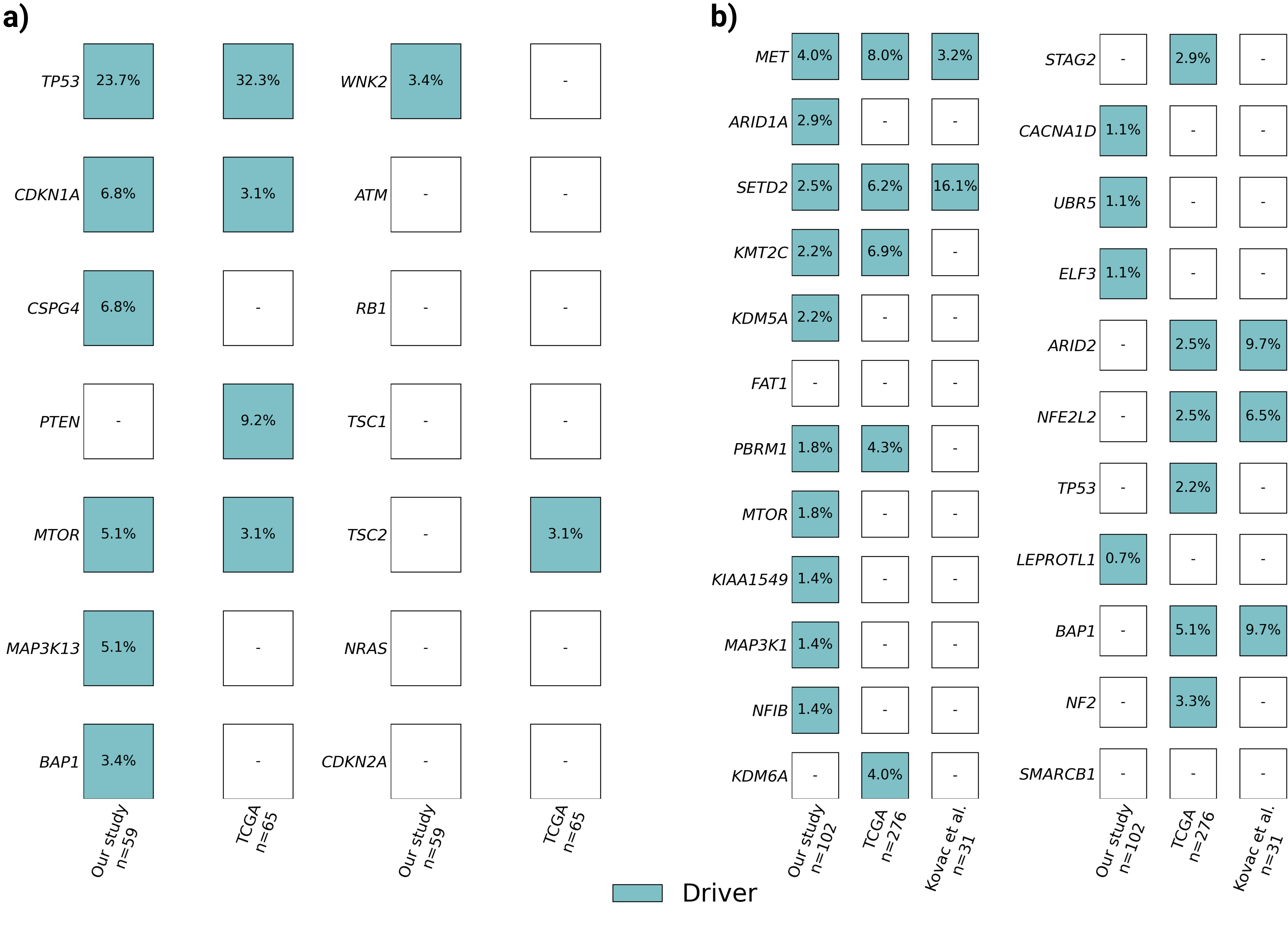

Supplement: S10 Figure [file EMS212285-supplement-S10_Figure.png]

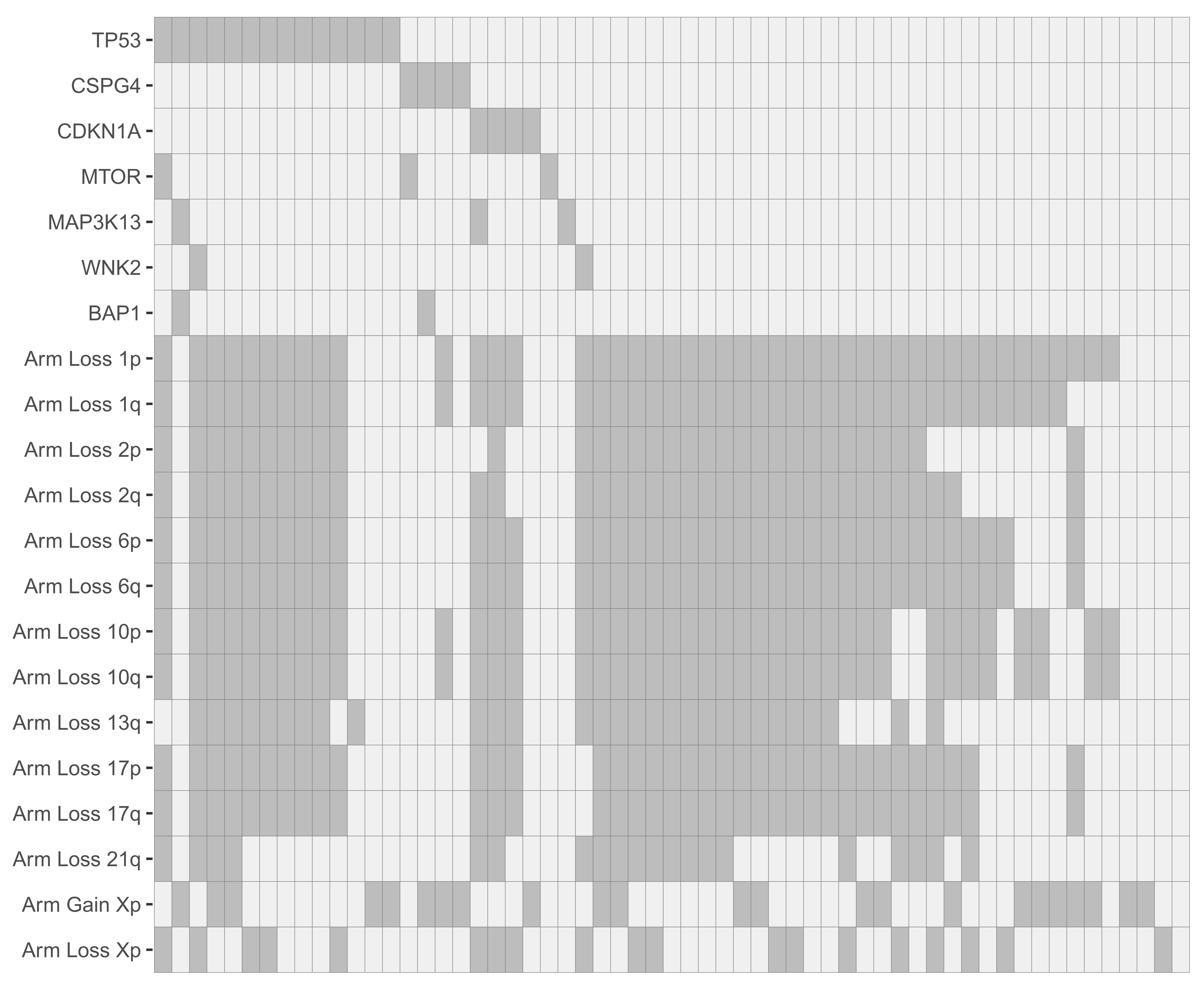

Supplement: S11 Figure [file EMS212285-supplement-S11_Figure.png]

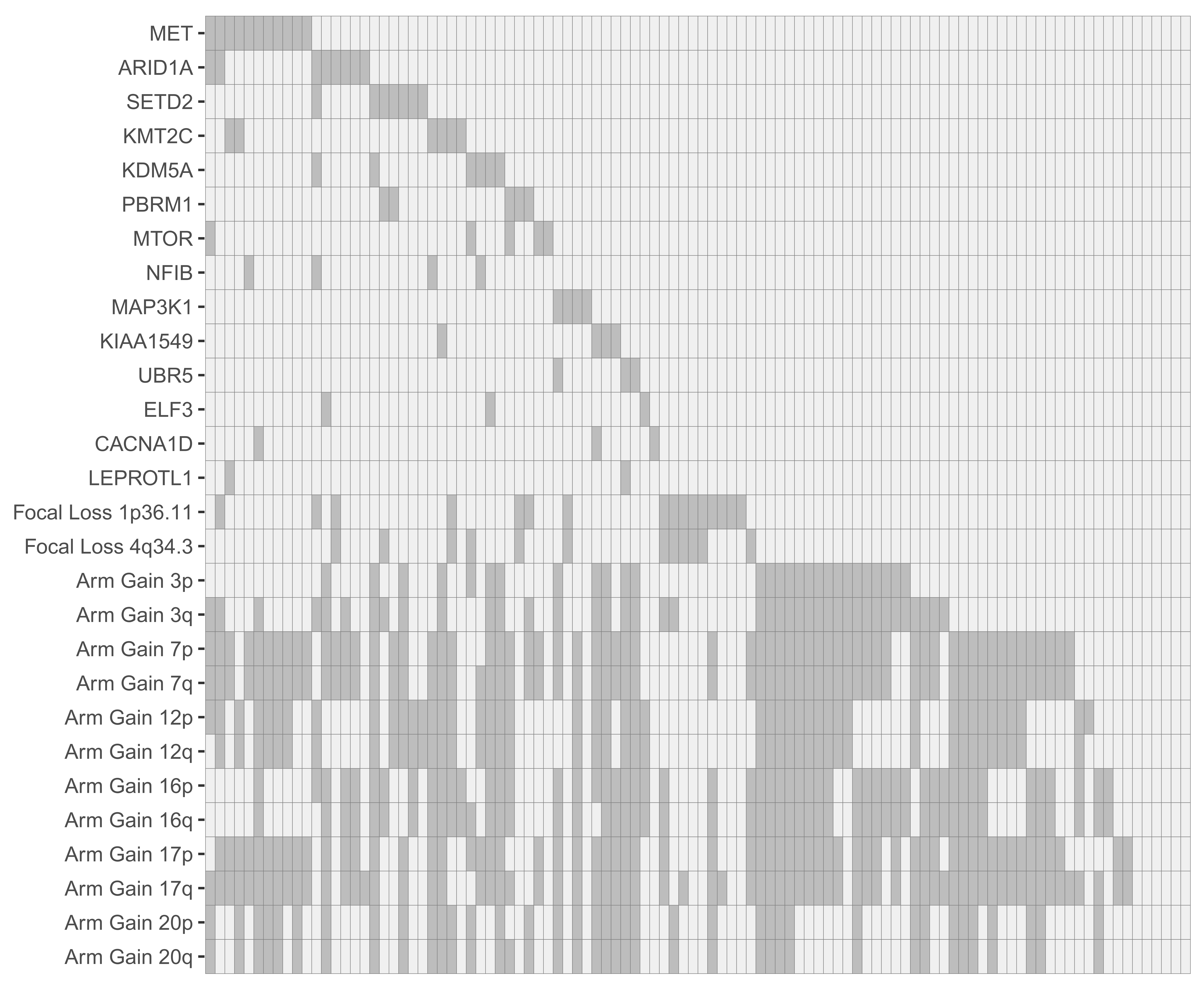

Supplement: S12 Figure [file EMS212285-supplement-S12_Figure.png]

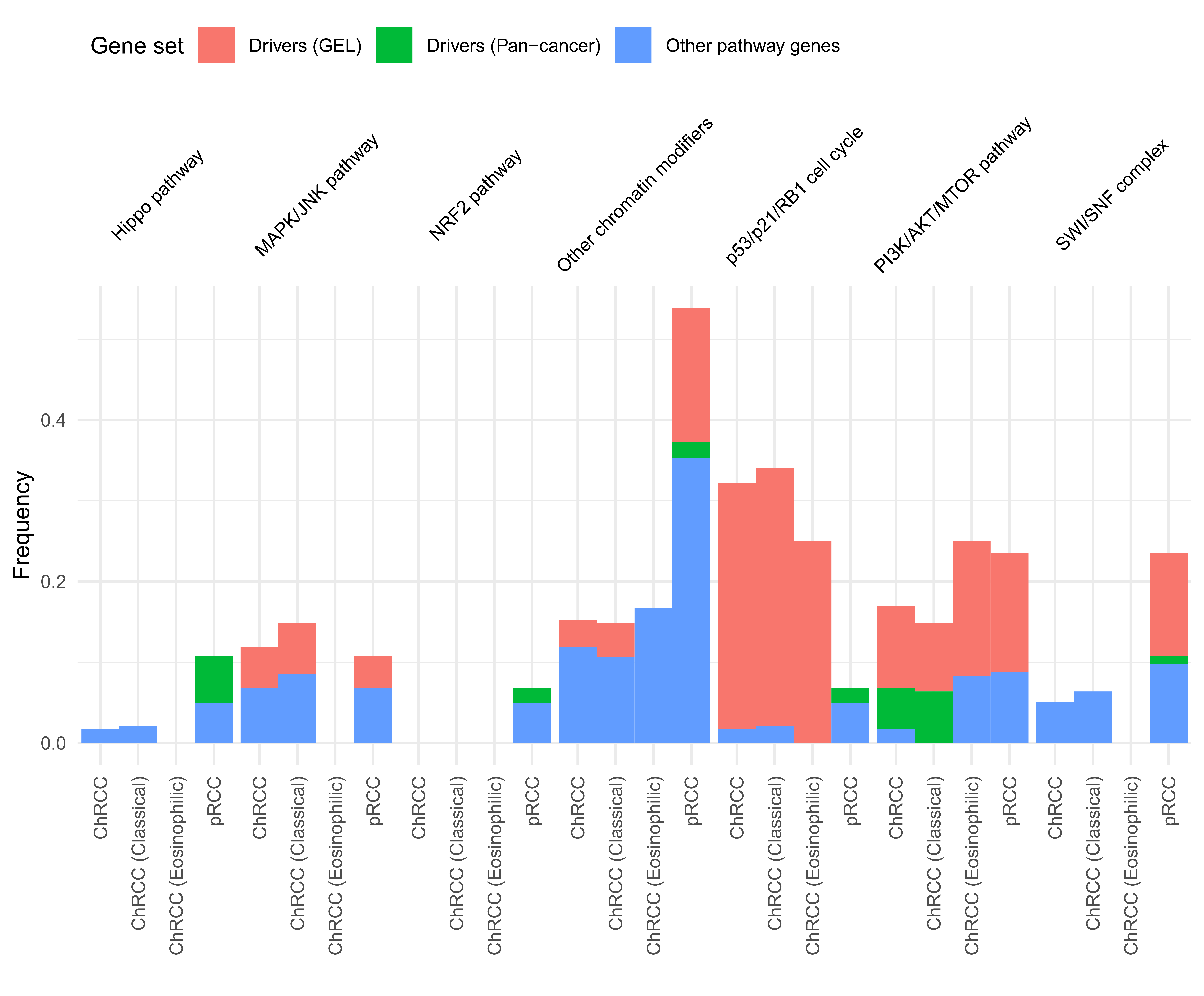

Supplement: S13 Figure [file EMS212285-supplement-S13_Figure.png]

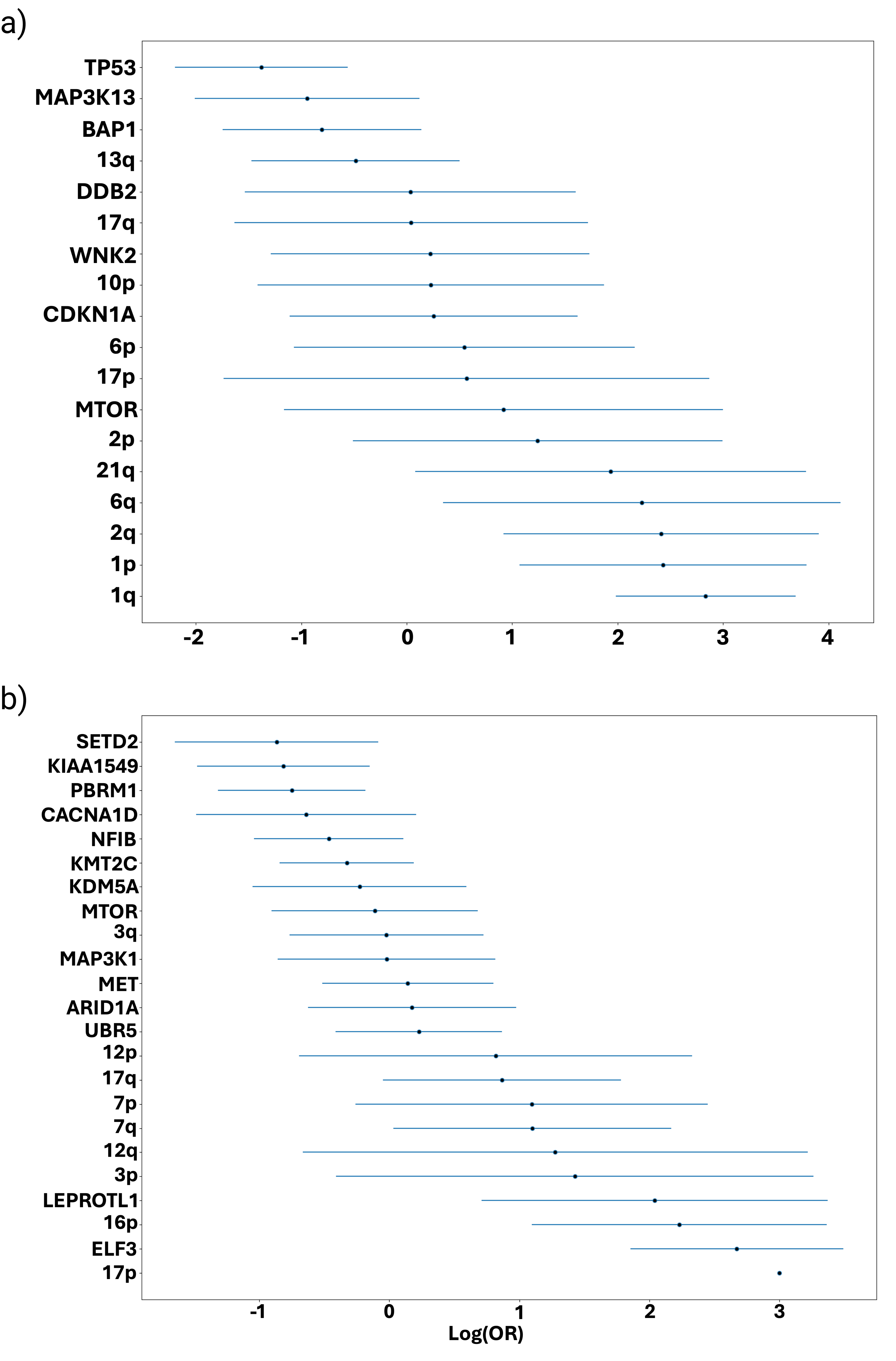

Supplement: S14 Figure [file EMS212285-supplement-S14_Figure.png]

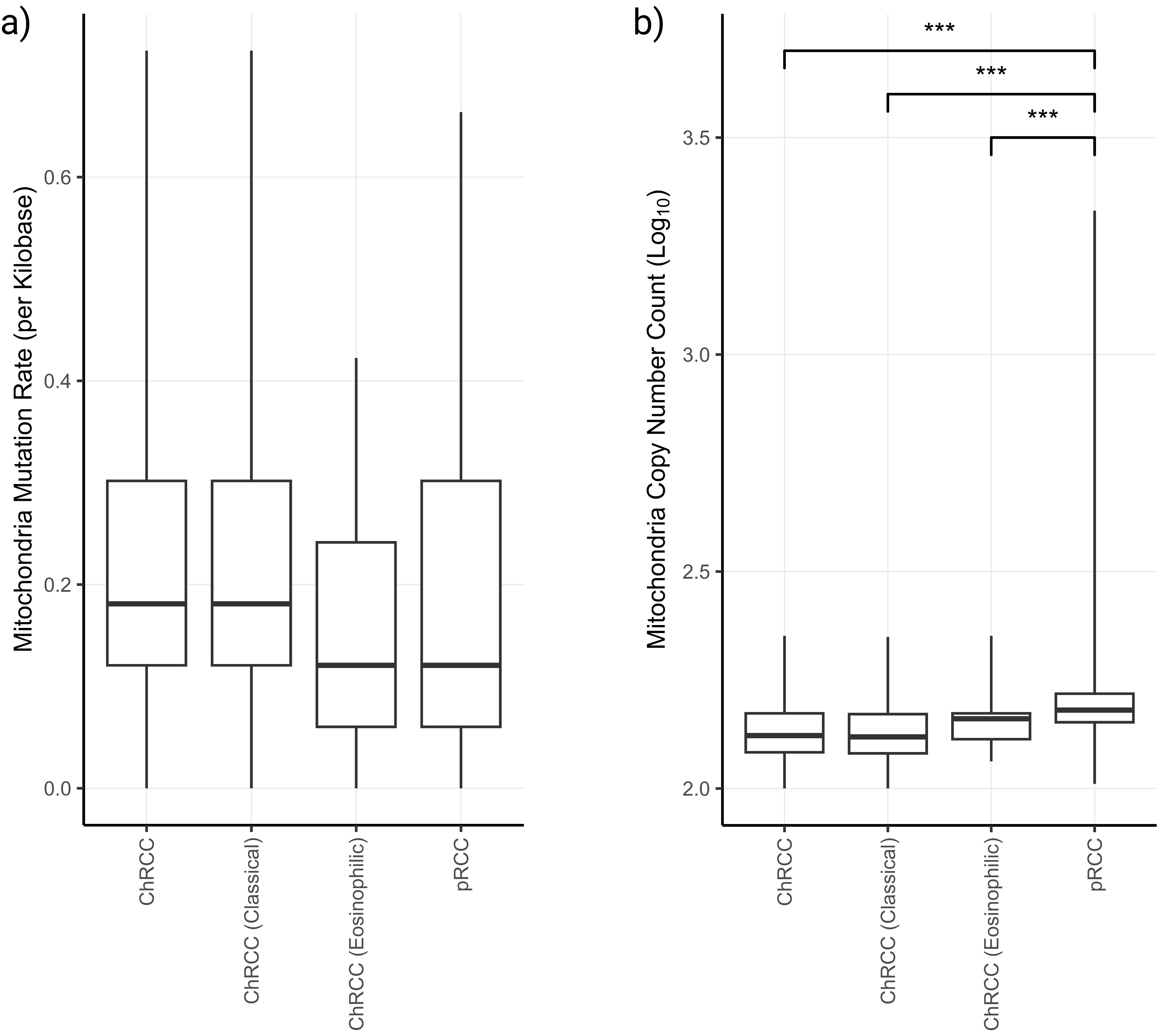

Supplement: S15 Figure [file EMS212285-supplement-S15_Figure.png]

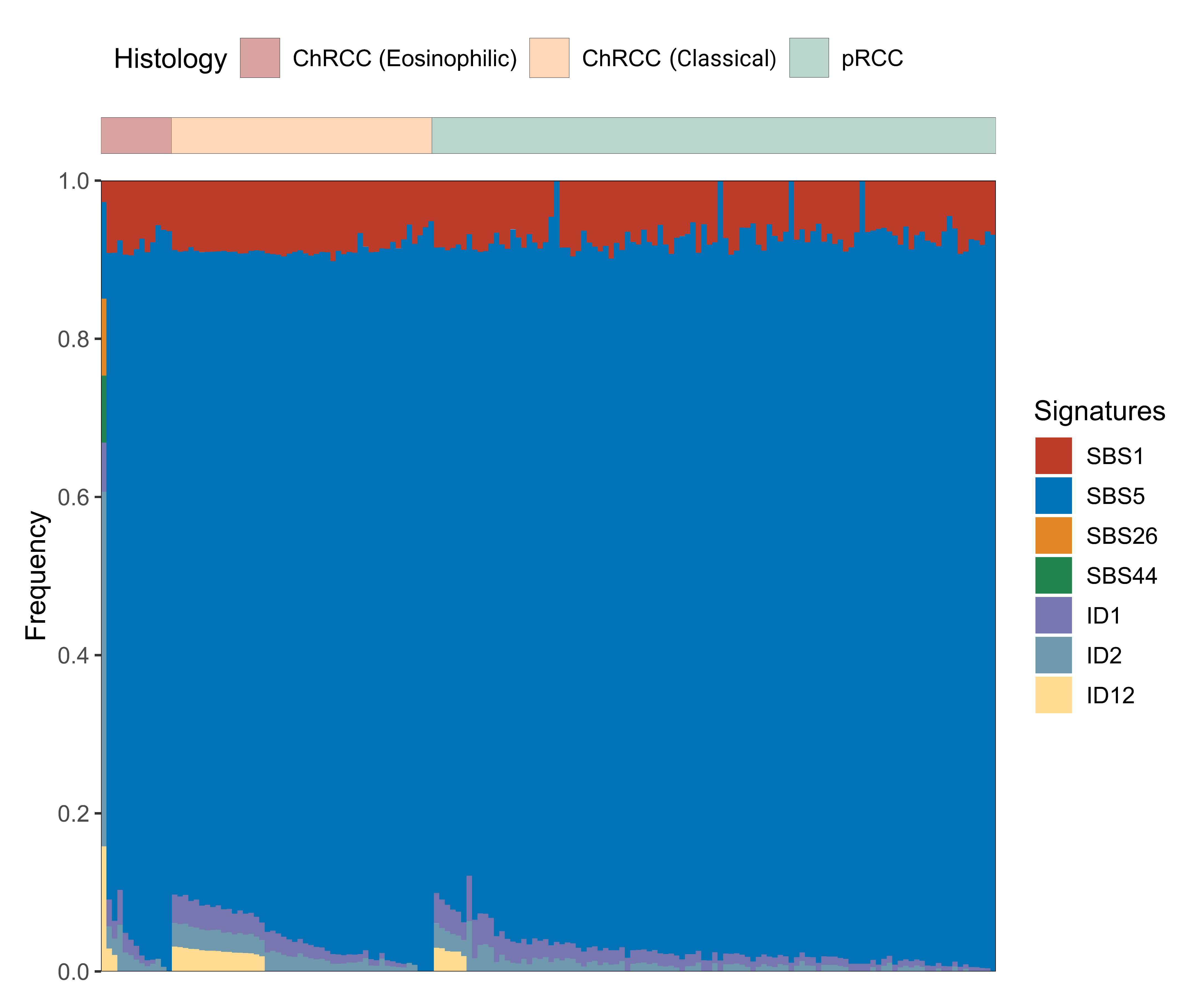

Supplement: S16 Figure [file EMS212285-supplement-S16_Figure.png]
