# Supplementary material for: Contrasting features of papillary and chromophobe renal cell carcinoma revealed by whole genome sequencing
Source: Mol Cancer Res. Author manuscript; Available in PMC 2026 Jun 15. (PMC13136883; doi:10.1158/1541-7786.MCR-25-0616)
Supplement: Supp Methods [file EMS212285-supplement-Supp_Methods.pdf]

SUPPLEMENTARY METHODS

1. SAMPLE COLLECTION 3

2. WHOLE GENOME SEQUENCING AND SOMATIC VARIANT CALLING 3

2.1. Genome sequencing and alignment 3

2.2. Single nucleotide variant and indel calling 4

2.3. Removing alignment bias introduced by semi-aligned-read soft-clipping 5

2.4. Evaluating microsatellite instability 5

2.5. Copy number alteration profiling 5

2.5.1. Stage 1: Initial profiling of copy number alterations 5

2.5.2. Stage 2: Evaluation of profile concordance with variant allele frequency distributions 6

2.5.3. Stage 3: Quality assessment 7

2.5.4. Stage 4: Re-profiling of copy number alterations 8

2.6. Structural variant calling 8

2.7. Whole genome duplication classification 9

2.8. Estimation of telomere length 9

2.9. Identification of extrachromosomal DNA 9

3. CLINICAL DATA 9

4. SAMPLE SELECTION 10

5. SINGLE NUCLEOTIDE VARIANT AND INDEL DRIVERS 11

5.1. Variant Effect Prediction 11

5.2. Identification of coding drivers 11

5.2.1 Input mutation pre-processing 11

5.2.2 Identification of coding drivers 11

5.3. Functional annotation of drivers and clinical actionability 13

5.4. Pathways 14

5.5. Non-coding drivers 15

6. SOMATIC COPY NUMBER ALTERATION PATTERNS 15

6.1. Copy number alteration classification 15

6.2. Enrichment of copy number alterations 16

6.2.1 Preparing GISTIC input, Initialization and Result 16

6.2.2 Annotation of CNA hotspots 16

7. SOMATIC STRUCTURAL VARIATION PATTERNS 17

7.1. Classification of simple and complex structural variants 17

7.2. Simple structural variation hotspots 18

7.2.1 Evaluating relationships between genomic features and SV rates 18

7.2.2 Simulating SVs 19

7.2.3 Identifying SV hotspots 19

7.3. Classification of SV hotspots as fragile sites 20

8. MUTATIONAL PROCESSES 21

8.1. Characterising mutational signatures 21

8.2. Homologous recombination deficiency 21

9. Mutation Timing 22
9.1 Timing of copy number alterations and somatic mutations 22
9.2 Relative ordering of driver events 22
10. Mitochondria Profiling 23
10.1 Somatic variant calling of the mtDNA genome 23
10.2. Mitochondrial copy number calling 23
10.3. Mitochondria driver discovery 23
11. Immune Profiling 24
11.1. Human Leukocyte Antigen (HLA) Typing and TCRA T-cell fraction 24
11.2 Neoantigen Prediction 24
11.3 Immune Escape 24
12. Correlating Clinicopathological And Mutational Variables 26
12.1 Correlations with Mutational Attributes 26

Source of each software package and external downloaded data is shown in **Supplementary Table S4**.

1. Sample collection

High-throughput sequencing data for tumours and germline samples were sourced from the 100,000 Genomes Project (100kGP, RRID: SCR_010502), an NHS initiative (1,2). Patient recruitment was coordinated by 13 Genomic Medicine Centres (GMCs) and associated hospitals throughout the UK. The renal tumour cases in this study were standard surgical cases, as reported by diagnostic histopathologists at the participating centres. Histology of renal cell carcinoma (RCC) was defined according to the diagnostic criteria outlined in the 5th edition of the WHO Classification of Urinary and Male Genital Tumours(3). All patients provided written informed consent. The collection of tissue and the preparation, extraction and quantification of DNA was undertaken locally, followed by transfer of DNA to a central biorepository. Illumina conducted whole genome sequencing of paired tumour/normal DNA. Processed BAM files were subsequently sent to Genomics England, where further quality checks were carried out, and were responsible for data storage.

2. Whole genome sequencing and somatic variant calling

2.1. Genome sequencing and alignment

Sequencing data for primary chromophobe RCC (ChRCC) and papillary RCC (pRCC) samples were sourced from the 100kGP Renal Cancer Domain main program, version v14 release (4). Sample preparation was conducted using Illumina TruSeq DNA PCR-free library preparation kit, and sequencing was performed on the HiSeq X, producing 150 base pair (bp) paired-end reads. Analysis was restricted to whole genome sequencing (WGS) data on fresh frozen and PCR-free samples to ensure low-input fast workflow, uniform coverage, and high sequencing accuracy (minimizing potential bias artifacts caused by PCR duplicates, particularly in GC-rich regions). Blood samples (serving as the source of the patients germline) and tumor samples were sequenced to average depths of 30x and 100x, respectively.

Samples with suboptimal sequencing quality were identified and removed using principal components analysis, based on:

- AT/CG dropout.
- Percentage of mapped reads.
- Percentage of chimeric DNA fragments.

- Average insert size.
- Uneven local coverage.

Samples classified as poor sequencing quality were not included in the 100kGP main programme v14 release and were therefore not considered in further. The initial sequencing analysis was performed using Illumina's North Star pipeline (v2.6.53.23). Sequence alignment to the Homo sapiens GRCh38Decoy assembly was completed using Isaac (RRID: SCR_012772, v03.16.02.19; ref 5).

2.2. Single nucleotide variant and indel calling

Strelka (RRID: SCR_005109, v2.4.7; ref 6) called single nucleotide variants (SNVs) and small insertions and deletions (indels). In addition to the default Strelka filters, the following filters were applied to exclude variants:

- Variants with a known high population germline allele frequency (≥1%) based on the gnomAD and/or 100kGP datasets (7).
- Variants with excessive somatic frequency (≥5%) in cancer, based on 100kGP dataset. This 5% threshold was determined based on the frequency of recurrent non-synonymous variants in hallmark genes listed in the Cancer Gene Census (8).
- Variants categorized as simple repeats by Tandem Repeats Finder (RRID: SCR_005659; ref 9).
- Indels for which ≥10% of base calls within a 50-base window on either side were filtered due to high sequencing noise.
- Overlapping 150bp reads where the majority map to multiple loci were excluded.
- SNVs which showed statistical evidence of having their corresponding ratio of tumour allele depths being different to their respective ratio of allele depths (via Fisher's exact test) at this site in a panel of normal samples (PoN). Only individuals who did not carry the alternate allele at a specific site were included in the allele depth counts. The PoN comprised 7,000 non-tumour genomes from the 100kGP, with PoN allele depths counted using the BCFtools mpileup function (RRID: SCR_005227, v1.9). To align with Strelka's preset filters, duplicate reads were removed, and quality thresholds were set with a base quality ≥5, mapping quality ≥5, and a phred score <80 (Fisher's exact test). The phred score threshold was selected to optimize precision and recall from a TRACERx truth set (10).

2.3. Removing alignment bias introduced by semi-aligned-read soft-clipping

The soft clipping of semi-aligned reads by Isaac results in the loss of support for alternative alleles located within five bases of the ends of each read. To address the allelic bias caused by this clipping, FixVAF (RRID: SCR_027746; ref 11) was applied to remove them. FixVAF soft clips all reads by five bases at both ends, irrespective of whether the bases are variant sites or whether the reads support a reference or alternate allele. Additionally, reads that support small insertions or deletions at the variant position are not considered.

2.4. Evaluating microsatellite instability

mSINGS (RRID: SCR_027728; ref 12) was utilized to detect tumours exhibiting microsatellite instability (MSI). Background models were created using the procedure outlined (https://github.com/sheenamt/msings/blob/master/Recommendations_for_custom_assays).
Microsatellite sites were generated with MISA (RRID:SCR_010765; ref 13), and only those overlapping regions with acceptable mappability were considered. Sites were excluded if they were found to be unstable in >5 microsatellite stable (MSS) test tumours or if they were not unstable in any test MSI tumours. After the background model was validated, mSINGS was applied to the tumour samples.

2.5. Copy number alteration profiling

Clonal and subclonal copy number alterations (CNAs) were called using the following four-stage iterative (**Supplementary Fig. S1-2**):

2.5.1. Stage 1: Initial profiling of copy number alterations

Battenberg (RRID: SCR_017098, v2.2.8; ref 14) was used to detect clonal and subclonal CNAs, as well as to estimate sample purity and tumor ploidy. Read counts for both reference and alternate SNV alleles were summed from tumor and normal samples. Heterozygous SNPs were phased with SHAPEIT2 (RRID: SCR_024335, v2.r904; ref 15), and A and B alleles were designated. The data were then segmented using piece-wise constant fitting (16), with subclonal copy number segments detected via t-tests. Sample purity and tumor ploidy were estimated using the method outlined by Van Loo *et al*. (17). Since the sequencing data were aligned to hg38, SNP positions were first converted to hg37 for phasing, and the resulting segments were then converted back to hg38.

2.5.2. Stage 2: Evaluation of profile concordance with variant allele frequency distributions

The expected frequency of variant alleles is influenced by several factors, including the proportion of tumor cells harboring the variant, the tumor's copy number profile, the number of chromosome copies carrying the variant (multiplicity), and the sample's purity (18). Given the tumor copy number profile and an estimated sample purity, the enrichment of variants that approximate at certain allele frequencies, which correspond to variants present in all tumor cells, is expected (17). If such enrichment is not observed, it could indicate inaccuracies in the copy number profile or sample purity. To address this, Battenberg output was validated by examining the SNV variant allele frequency (VAF) distributions.

For the analysis of SNV VAF distributions, only autosomal genome segments with copy number states of 1:1, 1:0, 2:2, 2:1, and 2:0, and no evidence of subclonal copy number alterations, were considered. These five copy number states were assessed individually, as the variant multiplicities and the expected clonal SNV VAFs differ between them (17). Copy number states corresponding to genome regions containing fewer than 5% of all SNVs were excluded from the analysis.

Expected locations of VAF distribution peaks were computed as:

$$\frac{\rho_{Battenberg}m}{2(1-\rho_{Battenberg})+\rho_{Battenberg}\psi_v} \quad (1)$$

where $\rho_{Battenberg}$ is the sample purity estimated by Battenberg, m the variant multiplicity (which can equal 1 or 2 in copy number states of 2:2, 2:1 and 2:0, and only 1 in states of 1:1 and 1:0), and ψ_v the ploidy of the tumour at the variant site.

The peaks of the VAF distribution were identified using kernel density estimation, implemented in peakPick (RRID:SCR_027740, v0.11; ref 19), with peaks corresponding to densities <0.3 being excluded. For each copy number state, the expected peak corresponding to the highest variant multiplicity was matched to the observed VAF distribution peak with the highest VAF. Any other expected peak locations were matched with the observed peak having the closest VAF. Tumor heterogeneity may complicate the detection of VAF peaks, so in cases where ≥1 expected peak locations were considered, the expected peak farthest from the observed peak (in terms of VAF) was discarded. Sample purity (ρ_i) was re-estimated for each remaining expected peak location (with VAF a) using the matched observed peak VAF:

$$\rho_i = \frac{2a}{m+\omega(2-\psi_s)} \tag{2}$$

where ω is the VAF of the matched observed peak and ψ_s the ploidy of the respective copy number state.

Afterwards, a re-estimated purity (ρ_{new}) was applied as the weighted average of the peak-wise purity estimates and used when samples were re-profiled:

$$\rho_{new} = \sum_i \frac{n_i \rho_i}{N q_i} \tag{3}$$

where n_i is the number of SNVs in genome regions of the respective copy number state, q_i is the number of variant multiplicities for the respective copy number state and *N* is the number of SNVs in genome regions of all copy number states in the analysis. The weighted average of the difference between the purity estimated by Battenberg and the peak-wise purity estimates was used to assessing CNA profile quality (see section 2.5.3):

$$\eta = \sum_i \frac{n_i |\rho_i - \rho_{Battenberg}|}{N q_i} \tag{4}$$

2.5.3. Stage 3: Quality assessment

Multiple criteria were used to assess Battenberg output validity:

- η <5%, indicating VAF distribution peaks were at the correct locations.
- DPClust (RRID: SCR_027723, v2.2.8; ref 14) was applied to see if there existed 1) a clonal mutation cluster that contained ≥5% of all SNVs with a CCF of between 0.9 and 1.1, or 2) no "super-clonal" mutation clusters that contained ≥5% of all SNVs with CCFs >1.1.
- Where the genome was mostly tetraploid (2:2), as defined by Battenburg, it was checked if there existed a peak in the SNV VAF distribution in 2:2 regions corresponding to a variant multiplicity of 1.
- There existed no homozygous deletion >10Mb.

Any sample that satisfied all above criteria had their corresponding CNA profiles and new purity estimates used in subsequent analyses. Samples not satisfying at least one criterion were re-profiled (*i.e.* proceeded to stage 4).

2.5.4. Stage 4: Re-profiling of copy number alterations

For samples that failed stage 3, new purity (ρ_{new}) was estimated via stage 2, whilst a new ploidy (ψ_{new}) estimate was considered applying the method by Van Loo *et al.* (17):

$$\psi_{new} = \frac{\rho_{Battenberg}\,(\psi_{Battenberg}-2)+2\rho_{new}}{\rho_{new}} \qquad (5)$$

These samples were re-profiled a maximum of three times using new re-estimated purity and ploidy each time. Any sample that continued to fail stage 3 checks after these three attempts were removed in subsequent analyses.

2.6. Structural variant calling

Somatic structural variants (SV) were called using a graph-based consensus approach applying Delly (RRID: SCR_004603, v0.7.8; ref 20), Lumpy (RRID: SCR_003253, v0.2.13; ref 21), Manta (RRID: SCR_022997, v0.28.0; ref 22) (all set with default parameters) and copy number alterations. Delly was run with post-filtering of somatic SVs using all normal samples. The collated SVs from each caller were further filtered if 1) the reads corresponding to the variant were found in the matched germline, 2) if tumour reads supporting the variant were <2%, or 3) if either of the two variant breakpoints were located in a telomeric or centromeric region or otherwise a non-standard reference contig (*i.e.,* not chromosomes 1-22, X or Y). All remaining SVs were combined with a modified version of PCAWG Merge SV, while allowing a 400bp window for breakpoint positions (23). The final set of SVs were then defined if at least two SVs callers had the SV, or if it was identified in a SV caller but additionally had a breakpoint within 3kb of a called CNA segment boundary (**Supplementary Fig. S3**).

Retrotransposition events are mechanistically distinct from other SV-generating events (24). Therefore SVs called by the graph-based consensus approach were categorised as likely retrotransposition events and excluded if: (i) xTea (RRID: SCR_027735; ref 25), which called somatically acquired long interspersed nuclear element (LINE-1) retrotransposition events, identified a transduced region in the same tumour sample within 10kb of either rearrangement break point, or (ii) xTea identified a transduced region within 10kb of either rearrangement breakpoint in ≥1% renal tumour samples. A 10kb threshold was used as most somatically acquired transductions span regions <10kb from a LINE-1 element (26). Alu elements, SINE-VNTR-Alu elements and processed pseudogenes were not called as together they comprise ≤3% of cancer retrotransposition events (24).

2.7. Whole genome duplication classification

To define the threshold used to classify each tumour as whole genome duplicated (WGD), the average genome copy number state (ψ_{ave}) was first calculated:

$$\psi_{ave} = \frac{\sum_{i=1}^{S}(L_i \sum_{j=1}^{2}(F_{j,i}(C_{j,i}^{Maj}+C_{j,i}^{Min})))}{\sum_{i=1}^{S} L_i} \quad (6)$$

where S is the number of copy number genome segments, $F_{j,i}$ is the fraction of tumour cells carrying copy number state j for genome segment i, $C_{j,i}^{Maj}$ and $C_{j,i}^{Min}$are the major and minor allele copy numbers for state j for genome segment i, and L_i is the base pair length of genome segment i. The parameters $F_{1,i} = 1$ and $F_{2,i} = 0$ If there was no evidence of a subclonal alteration. WGD classification was calculated via the approach by Gerstung *et al.* (27):

$$\{WGD,\ if\ 2.9 - 2H < \psi_{ave}; Not\ WGD,\ otherwise\} \quad (7)$$

where H is the fraction of the respective genome where loss of heterozygosity occurred (minor allele copy number = 0).

2.8. Estimation of telomere length

Germline and tumour telomere lengths were estimated from their respective bam files using TelomereCat (RRID: SCR_027747, v3.3.0; ref 28) with default parameters applied.

2.9. Identification of extrachromosomal DNA

Amplicon structures were identified using AmpliconArchitect (RRID: SCR_023150, v1.2; ref 29), using default parameters. Amplicons were classified with AmpliconClassifier (RRID: SCR_023150, v0.3.6; ref 30) and cyclic amplicons were inferred to represent extrachromosomal DNA as recommended.

3. Clinical data

Demographic and clinical information was collected from various sources, including the Genomic Medicine Centres (GMC; https://www.england.nhs.uk/genomics/nhs-genomic-med-service/), NHS Digital (NHSD; https://digital.nhs.uk/), and the Public Health England's National Cancer Registration and Analysis Service

(PHE-NCRAS; https://www.gov.uk/guidance/national-cancer-registration-and-analysis-service-ncras). Tumour pathology reports were extracted via GEL. Tumour samples were matched with their corresponding PHE-NCRAS records by using tumour sampling dates and PHE-NCRAS treatment dates, allowing a maximum discrepancy of seven days.

Data extracted for all samples included information on sex, cancer diagnosis date, tumour stage, tumour sampling date, age at the time of tumour sampling, last reported clinical follow-up date, survival outcome, and date of death if applicable. ChRCC tumours were not graded due to the Fuhrman nuclear grade lacking compatibility with ChRCC molecular features (31,32).

4. Sample selection

213 fresh-frozen PCR-free ChRCC and pRCC primary tumour samples were selected (see section 1). Participants were filtered out if they met one of the following criterion (**Supplementary Table S1**):

- Age of the Participant during tumour sampling was unavailable.
- Participant was <18 years old during tumour sampling.
- Participant sex inferred from sequencing data did not match sex reported by the submitting GMC.

One participant was excluded due to a conflict in reported sex. To ensure precision and sensitivity of variant calling (6) is adequate (inadequate calling could be due to low sample purity (18,33) or other sequencing quality deficiencies), tumour and germline samples for each participant were excluded based on the following:

- Tumour sample cross-contamination >1%.
- Cross-contamination of the matched germline sample with another germline sample was >1%.
- The majority of called SNVs had low VAFs (median SNV VAF<0.1).
- If <500 SNVs were called, with the threshold based on clear cell RCC in PCAWG (34).

A total of 20/213 participants were excluded for failing at least one of the variant calling criteria (**Supplementary Table S1**). A total of 22/213 tumour samples had CNA profiles that failed CNA QC (see section 2) and were excluded. Some participants had multiple tumour samples sequenced, extra duplicates representing 17/213 tumour-normal pairs, thus only the tumour sample with the highest purity in the primary analysis was considered. This resulted in a cohort of 61 primary ChRCC and 103 primary pRCC matched tumour-normal samples (**Supplementary Table S1**).

5. Single nucleotide variant and indel drivers

5.1. Variant Effect Prediction

To run driver-identification programmes and to identify the consequences for all somatic variants, Variant Effect Predictor (VEP) built on Ensembl (RRID: SCR_001630, v101 Grch38, McLaren *et al.*; ref 35) annotated each variant using the commands: *vep -i --assembly GRCh38 –no_stats –cache –offline –symbol –protein -o --vcf –canonical –dir --hgvs –hgvsg –fasta --plugin CADD, --plugin UTRannotator*. Mutations were mapped to their Ensembl canonical protein-coding transcripts. Non-synonymous mutations as any SNV or indel classified as having a moderate or high calculated consequence as stated by VEP. SNVs and indels were assigned a Combined Annotation Dependent Depletion (CADD, RRID: SCR_014886, v1.6) score using the VEP plugin (https://github.com/kircherlab/CADD-scripts) (36–38). The UTRannotator plugin (RRID: SCR_027734; ref 39) annotated all five prime untranslated regions (5' UTR).

5.2. Identification of coding drivers

The Integrative OncoGenomics pipeline (IntOGen, RRID: SCR_027727, downloaded February 2021) was used to identify an initial set of candidate protein-coding driver genes (Section 5.2.2), with additional filtering steps applied to remove false positives (Section 5.3).

5.2.1 Input mutation pre-processing

Hypermutated tumour samples for each cohort were excluded from driver gene identification, based on either having either >10,000 mutation or had an outlier mutation count (upper quartile + 1.5 × interquartile range) compared to the rest of its corresponding cohort. This led to 2/61 and 1/103 of the ChRCC and pRCC tumour samples respectively being excluded from the downstream driver analysis. Otherwise, mutations in the Hartwig Consortium (41) (i.e, PoN) were also excluded when applying each driver gene identification methods.

5.2.2 Identification of coding drivers

The set of driver gene algorithms prioritise different mutational features of each matched tumour-normal when selecting candidate driver genes, and included the following:

1. dNdSCV (RRID: SCR_017093; ref 42) selects drivers under positive selection via the ratio of nonsynonymous to synonymous mutations (after correction for local trinucleotide context).
2. OncodriveFML (RRID: SCR_027731; ref 43) identifies drivers with mutations that have a higher functional impact (based on CADD Scores(36–38); Section 5.1) than expected.
3. OncodriveCLUSTL (RRID: SCR_027730; ref 44) identifies drivers with a higher concentration of mutations than expected compared to a simulated nucleotide context-based background model.
4. cBaSE (RRID: SCR_027765; ref 45) identifies driver genes under positive selection based on per-gene probabilities of missense and nonsense mutations, and compares said probabilities to a simulated neutral mutation model.
5. MutPanning (RRID: SCR_027729; ref 46) identifies positive selection of drivers which exhibit mutations with sequence contexts that greatly deviate from the contexts of mutations within a set window.
6. HotMaps3D (RRID: SCR_027725; ref 47) detects the driver gene has a cluster enrichment of mutations considering a 3-dimensional protein structure context. Protein structures are from The Protein Data Bank (PDB, RRID: SCR_012820, downloaded March 2020; ref 48).
7. smRegions (RRID: SCR_027733; ref 49) detects higher enrichment of nonsynonymous mutations in specified regions, where protein domains are considered in this analysis. Protein family (pfam) domains were mapped to Ensembl canonical transcripts.

The output of the seven driver gene identification methods was combined using IntOGen's weighted ranking system. First the top-40 ranked genes and their corresponding *P*-values in each of the seven driver identification methods were defined. Then, the lists of per-method ranks were combined using Schulze's voting method to generate a single "consensus" ranking that takes into account how well each method gives precedence to the top genes in a "truth set" (created from any Tier 1 or Tier 2 somatically mutated genes in the COSMIC Cancer Gene Census (CGC, RRID: SCR_002260, v92; ref 50). The corresponding *P*-values were combined using a weighted Stouffer *Z*-score, followed by two sets of adjusted Stouffer *Q*-values created (one set adjusted the *P*-values corresponding to all genes and the other considering CGC genes only). Driver candidates were then assigned the following tiers:

- Tier 1 candidate drivers have a Schulze's voting consensus ranking higher than first gene in the ranking with a corresponding Stouffer $Q > 0.05$.
- Tier 2 candidate drivers are non-tier 1 CGC genes with Stouffer CGC $Q < 0.25$, with the consensus ranking criteria not considered.
- Tier 3 candidate drives are non-tier 1/2 genes with a Stouffer $Q < 0.05$.

- Tier 4 have Stouffer *Q* >0.05.

Candidate driver genes were excluded if they met the following criteria:

- Categorised as Tier 4.
- Only statistically significant (Q <0.1) in one driver discovery method.
- TCGA data reports candidate drivers as having very low or no expression in cohort.
- Candidate driver is classified as an olfactory receptor gene, known artefact or long gene (e.g., TTN).
- Candidate driver has a nonsynonymous SNV with corresponding strand bias ≥10 (Strelka Info field SNVSB) suggesting a false positive.

A candidate gene may be marked with one of the following annotations for further investigation:

- Candidate driver is a non-tier 1 CGC candidate gene with ≥1 mutations per sample and oe_syn/ms/lof > 1.5 based on GnomAD (RRID:SCR_014964, v2.1; ref 51) constraint metric estimates (GERMLINE).
- Candidate driver has ≥3 mutations in ≥1 tumour and is a non-CGC gene (SAMPLE_3_MUTS).
- Candidate driver has no literature annotations according to CancerMine (RRID: SCR_027748; ref 52) and is a non-CGC gene (LITERATURE).

Otherwise all other candidates drivers are set as passing the pipeline.

Candidate driver roles were assigned assigned a predicted role (oncogene or tumour suppressor gene (TSGs)) using dN/dS ratios corresponding to their missense (w_{mis}) and nonsense (w_{non}) mutations derived from dNdSCV (https://bitbucket.org/intogen/intogenplus/src/master/core/intogen_core/postprocess/drivers/role.py). A "distance" metric was calculated by

$$distance = \frac{(w_{mis} - w_{non})}{\sqrt{2}} \qquad (8)$$

and this metric assigned the candidate driver role as

- Oncogenes if distance >0.1, due to excess of missense to nonsense mutations.
- TSG if <0.1, due to an excess of nonsense to missense mutations.
- Ambiguous otherwise.

All driver candidates were annotated by their overlap with any IntOGen cohorts from a previous IntOGen pan-cancer analysis (01 Feb 2020) as well as from a pan-cancer TCGA analysis (53).

5.3. Functional annotation of drivers and clinical actionability

Nonsynonymous mutations were additionally annotated using the OncoKB API (RRID: SCR_014782, v3.11; ref 54) where the HGVSg identifier (or otherwise, a combination of gene symbol, consequence and HGVSp)

was used to map mutations to OncoKB annotations. Nonsynonymous mutations were annotated as oncogenic if:

- The mutation is classified as “Oncogenic”, “Likely Oncogenic” or “Predicted Oncogenic” by the OncoKB API.
- The mutation is missense and is recurrent (≥3 samples).
- The mutation is within a TSG or ambiguous driver and is classified as a protein-truncating mutation (splice acceptor, splice donor, frameshift, stop lost, stop gained or start lost).

Mutations were classified as variants of uncertain significance (VUS) otherwise if they did not meet above criterion.

We further assessed the clinical actionability of driver gene mutations by interrogating OncoKB Knowledge Base (RRID: SCR_014782, v3.11; ref 54). Treatments annotated in OncoKB are segregated by the following clinical categories,

- Level 1 - FDA-recognised biomarker predictive of response to an FDA-approved drug in this condition.
- Level 2 - Standard care biomarker recommended by the NCCN or other professional guidelines predictive of response to an FDA-approved drug in this indication.
- Level 3 - Compelling clinical evidence supporting the biomarker as being predictive of response to a drug in this indication/Standard care or investigational biomarker predictive of response to an FD-approved or investigational drug in another indication.
- Level 4 - Compelling biological evidence supporting the biomarker as predictive”.

The COSMIC Mutation Actionability in Precision Oncology database (RRID: SCR_002260; ref 8) was also examined, with annotated treatments classified as,

- Level 1 - Approved marketed drug with demonstrated efficacy at the mutation.
- Level 2 - Phase 2/3 clinical results meeting primary outcome measures.
- Level 3 - Drug in ongoing clinical trials.
- Level 4 - Case studies.

5.4. Pathways

Cellular pathways containing identified coding/non-coding CHRCC and pRCC driver genes (**Supplementary Table S9-10**) were initially found via PubMed literature search. Afterwards, the set of pathways were further

validated via ActivePathways (RRID: SCR_027736, v1.1.1; ref 55), which searches across canonical pathway curated gene sets, gene ontology gene sets, oncogenic signature gene sets (for example, Kegg curated pathways), and Reactome pathway curated gene sets from MSigDB (RRID: SCR_016863, v7.5.1; refs 56,57).

5.5. Non-coding drivers

Non-coding drivers were considered in the following genomic regions referenced by Ensembl (RRID: SCR_001630, v101; ref 58):

- Core promoters, <200bp downstream from the transcriptional start site (TSS) and <50bp upstream of the TSS of canonical protein-coding transcripts (n=19,283).
- Distal promoters, <2kb upstream of the TSS of canonical protein-coding transcripts (n=19,296).
- 5'UTRs of canonical protein-coding transcripts (n=18,613).
- 3'UTRs of canonical protein-coding transcripts (n=18,806).
- Non-canonical splice regions being any region that extends 30bp into the intron from essential splice donor or acceptor sites of canonical protein-coding transcripts (n=18,163).

Any coding sequences (CDS) or protein coding exon regions were excluded from the regions of interest using BEDOPS (RRID: SCR_012865, v2.4.39; ref 59). Positive selection in non-coding regions was assessed using:

- OncodriveFML (RRID: SCR_027731; ref 43) with the "indel-max" argument so observed indels are classified as a set of SNVs with the corresponding functional impact score being the maximum of the substitution set.
- ActiveDriverWGS (RRID: SCR_027737; ref 60) inspects if a region has a higher mutational burden than expected, given mutational burden around local background sequence, applying Poisson generalised linear regression modelling.

6. SOMATIC COPY NUMBER ALTERATION PATTERNS

6.1. Copy number alteration classification

Copy number alterations (Section 2.5) were classified into six categories (**Supplementary Table S3**) depending on the copy number states and whether a genome was affected by whole genome duplication. If there existed intra-tumour heterogeneity in CNA states (i.e subclonal CNAs were present) then the largest cell fraction was defined as the primary state.

6.2. Enrichment of copy number alterations

6.2.1 Preparing GISTIC input, Initialization and Result

Genomic Identification of Significant Targets in Cancer (GISTIC, RRID: SCR_000151, v2.0.2.3; ref 61) was applied to identify recurrent arm-level copy number events, and focal copy number amplifications and deletions. The per-segment normalised copy numbers (SegCN) was calculated as a function of the major (nMaj) and minor (nMin) copy number states and WGD. SegCN was thresholded to a minimum of -2 and maximum of 2.

For non-WGD tumours, per-segment normalised copy number is defined as:

$$SegCN = (nMaj + nMin)-2 \tag{9}$$

however for non-WGD tumours from males, the X chromosome per-segment normalised copy number is defined as:

$$SegCN = (nMaj+nMin)-1 \tag{10}$$

For WGD tumours, per-segment normalised copy number is defined as:

$$SegCN = [(nMaj+nMin)-4]/2 \tag{11}$$

where for WGD tumours from males, X chromosome per-segment normalised copy number is defined as:

$$SegCN = (nMaj+nMin)-2 \tag{12}$$

GISTIC was run using the following parameters: *-conf 0.99 -broad 1 -qvt 0.25 -genegistic 1 -gcm extreme -brlen 0.5 -rx 0 -twoside 1 -scent median -armpeel 1 -arb 1 -refgene hg38.UCSC.add_miR.160920.refgene.mat*

6.2.2 Annotation of CNA hotspots

Alterations from the broad analysis with $Q<0.05$ were taken to indicate recurrent arm-level events. Copy number segments comprising greater than 50% of the total chromosome arm length were defined as arm-level events.

Deletions refer to segments with homozygous deletions or loss of heterozygosity, while amplification refers to gains (small gains) or amplifications (big gains; **Supplementary Table 3**). In the case of subclonal CNAs, nMaj and nMin values corresponding to the largest cell fractions were used.

For focal events identified by GISTIC, the “wide region” was used to compare the potential extent of overlap with copy number segments. Segments were defined as overlapping focal events if either the segment interval comprised greater than 50% of the focal region, or vice versa, using pybedtools (RRID: SCR_021018; ref 62) and bedtools (RRID: SCR_006646, v2.3.0; ref 63).

The effect on drivers genes affected by either loci targeted with focal amplifications/deletions or arm level alterations was considered. A driver gene, or an implied driver gene from other studies, is annotated as being altered if,

1. The driver gene overlaps with commonly found genes at focal amplifications and deletions reported in a previous pan-cancer study that used GISTIC, considering genes identified as candidates by Zack *et al.* (64).
2. The driver gene had their established or predicted role consistent with the copy number change (oncogene with amplifications and tumour suppressor with deletions).

7. SOMATIC STRUCTURAL VARIATION PATTERNS

7.1. Classification of simple and complex structural variants

Using ClusterSV (RRID: SCR_027722; ref 25), all rearrangements were classified as

- Rearrangement footprints, being positionally associated rearrangement breakpoint sets, applying the string notation approach via Li, *et al.* (23).
- Rearrangement clusters being mechanistically related arrangements. and classified as simple or complex events if they comprised ≤2 or ≥3 individual rearrangements respectively.

Simple events were further classified using the corresponding string notations as either deletions, tandem duplications, balanced inversions, balanced translocations, unbalanced translocations or unclassified simple SVs.

Complex SVs were classified as chromothripsis events (65,66) if:

- The cluster did not meet the conditions to be defined as a chromoplexy event (see below).
- The cluster contained ≥2 interleaved intra-chromosomal rearrangements. This liberal threshold was used to reduce false negatives as it has been documented in RCC that interleaved clusters are fewer compared to other cancers (where in comparison a threshold of ≥6 is applied) (65).

- A contiguous series of ≥4 genome segments oscillating between 2 copy number states, or ≥5 genome segments oscillating between 3 copy number states, was present in the cluster.
- If the distribution of intra-chromosomal fragment-join orientations in the cluster showed statistically significant evidence of diverging from a multinomial distribution with equal probabilities for each of the four rearrangement orientation categories (deletion-like, duplication-like and head-to-head and tail-to-tail inversions; false discovery rate >0.2).

Otherwise, complex SVs were classified as chromoplexy events if:

- The cluster consisted of between 3 and 30 rearrangements.
- The rearrangements within the cluster spanned ≥3 chromosomes (23). To define SV chains between chromosomes, a graph-based approach was applied with nodes representing breakpoints and each node is connected via an edge if they fall within 1Mb of each other but are not involved in the same rearrangement.
- Balanced translocations, with a deletion bridge between break ends or no copy number change, represent ≥50% rearrangements within the cluster.

7.2. Simple structural variation hotspots

Hotspot regions for each type of simple SV (Section 7.1) were identified via permutation-based procedure as per Glodzik *et al.* (67). Hotspots for complex SVs were not considered.

7.2.1 Evaluating relationships between genomic features and SV rates

The following features (67) were correlated with SVs (using negative binomial regression):

- Average tumour total copy number.
- The presence of genes highly or lowly expressed. Categorization based on if said genes had corresponding RNA-Seq by Expectation-Maximization (RSEM) values in the top 25% and bottom 75% protein-coding genes respectively in TCGA samples.
- Guanine-Cytosine content within the GRCh38 reference genome.
- ALU repeats and other genomic repeats (via University of California Santa Cruz (UCSC) Genome Browser (RRID: SCR_005780; ref 68).
- Segmental duplications via the Segmental Duplication Database (69).

- Cellular replication timing defined by embryonic kidney cells (HEK293, Int57383924 from ReplicationDomain) (70).
- DNase peaks, with DNase-seq data obtained from female adult (47 years) kidney tissue from Encode (GRCh38, ENCAN876VFO, ENCODE4 v3.0.0-alpha.2; refs 71,72).
- Chip-seq H3K36me3 peaks (GRCh38, ENCAN946KXL, ENCODE4 v1.7.0) and H3K9me3 peaks (GRCh38, ENCAN127GOZ, ENCODE4 v1.7.0) in kidney tissue from a 50 years male adult from Encode (71,72).
- Fragile Sites defined by Bignell *et al.* (73).

Statistically significant features were selected to be part of SV simulation (Section 7.2.2).

7.2.2 Simulating SVs

Simulation of SVs comprised of the following steps:

- The genome was divided into non-overlapping 1Mb bins, and statistically significant correlated genomic features of each bin normalized to a mean of 0 and standard deviation of 1.
- Expected number of break ends in each 1Mb bin was estimated via a negative binomial regression model conditional on the features.
- SVs were simulated (while preserving number, length and type of the observed SVs) by first sampling a bin under probabilities proportional to the expected numbers of break ends in each bin. Each corresponding partner break end was then simulated by selecting the position either upstream or downstream (with equal probability) with the pairwise breakpoint distance being the same as the observed SV. It was resimulated if either break end fell within an uncallable region (*i.e.* a telomere or centromere).
- A null distribution of expected SVs for each of the 1Mb bins was generated by repeating the previous three steps a total of 1,000 times.

7.2.3 Identifying SV hotspots

Regions of the genome containing greater numbers of SV break ends than expected by chance were identified using piecewise constant fitting (PCF). PCF was applied, to the $\log_{10}$, of inter-mutational distances (IMD) between sorted successful breakend points of all SVs. To find hotspots, the observed (d_i^{obs}) and expected (d_i^{exp}) number of breakends per base pair for each PCF segment (i) was defined as:

$$d_i^{obs} = \frac{a_i}{s_i} \tag{13}$$

$$d_i^{exp} = \frac{\sum_{j=1}^{n} b_j}{s^{bin} n} \tag{14}$$

where a_i is the number of break ends in the segment, s_i is the length of the segment in base pairs, b_j is the expected number of SVs in bin j, n is the number of bins overlapping the segment, and s^{bin} is the bin size (in this case, s^{bin}=1Mb). Each PCF segment is assigned a simple SV enrichment factor (β_i^{simple}), defined as the observed-expected ratio of breakends per base pair:

$$\beta_i^{simple} = \frac{d_i^{obs}}{d_i^{exp}} \tag{15}$$

As the PCF algorithm uses parameters γ (that controls the smoothness of the segmentation) and k_{min} (the minimum number of mutations in a segment), they were set to $\gamma = 10$ and $k_{min} = 4$ respectively. The false discovery rates (FDRs) at each β^{simple} value were estimated by applying PCF to both the observed and simulated SV sets, which was followed by dividing the mean number of segments with a β^{simple} value at least as great in the simulated SV sets by the number of segments with a β^{simple} value at least as great in the observed SV set. Should CNAs not support any SVs in a hotspot region, then the SV hotspot was classified as an artefact and removed. If SV hotspots overlapped, they were merged into a single wider region.

7.3. Classification of SV hotspots as fragile sites

SV hotspots were annotated as overlapping fragile sites if at least three of the following six criteria were annotated:

- Hotspot region overlapped late replicating region (74), where late replication segments are defined if corresponding mean Repli-Seq values ≤0. Replication timing was based on embryonic kidney cells (HEK293, Int57383924) from ReplicationDomain (70).
- Hotspot region had low gene-density (≤5 per MB) (75).
- Hotspot region overlapped a gene greater than 300kb in size, where fragile sites are more likely to be located (76).

- If the majority of SVs within the hotspot region overlapped the gene of greatest size. A gene was classified as SV enriched if the ratio of SV break point densities in said gene compared with the breakpoints intergenic regions flanking 1Mb upstream and downstream was >5.
- Overlapped reported fragile sites obtained from NCBI or literature curation (73).
- Additionally if the hotspot region overlapped reported fragile sites from an analysis of the PCAWG cohort (34).

LiftOver (RRID: SCR_018160; ref 67) was used to map fragile site coordinates from the PCAWG study and NCBI/literature to the GRCh38 reference genome. SV hotspots classified as fragile were not considered further.

8. Mutational Processes

8.1. Characterising mutational signatures

Mutational signatures were extracted *de novo* using SigProfilerExtractor (RRID: SCR_023121; ref 77). Single-base-substitution (SBS), doublet-base-substitution (DBS) and insertion and deletion (ID) signatures were considered and were decomposed to known COSMIC signatures (RRID: SCR_002260, v3.2; ref 8). SBSs were considered with respect to their tri-nucleotide and transcriptional context (SBS288). All signatures were extracted using random initialization, 500 NMF replicates, and between 10,000 and 1,000,000 NMF iterations. We assumed the solution would lie in the range of 1 and 30 SBS and ID signatures, and 1 and 20 DBS signatures. Solutions used in downstream analysis were manually selected by considering the solution stability across NMF replicates, the error in mutational profile reconstruction, and concordance with previously reported renal cancer signatures (77,78).

8.2. Homologous recombination deficiency

Homologous recombination deficient (HRD) tumours were identified using HRDetect (RRID: SCR_027726; refs 79,80), making use of SBS3 and SBS8 activities, rearrangement signatures RS3 and RS5 activities, the proportion of deletions with microhomology and HRD index. SBS3 and SBS8 activities were estimated using SigProfilerExtractor (RRID: SCR_023121; ref 77), although SBS3 was not detected in any tumour. RS3 and RS5 activities were estimated using HRDetect, based on the rearrangement signatures characterised by Nik-

Zainal *et al*. (80). HRDetect was trained using breast cancers but it has been shown to perform well when applied to other cancers (79).

9. MUTATION TIMING

9.1 Timing of copy number alterations and somatic mutations

The relative evolutionary timing of SNVs and CNAs was estimated by MutationTimeR (RRID: SCR_027739, v0.99.3; ref 27). Briefly, MutationTimeR groups SNVs into four classes: early clonal, late clonal, subclonal, unspecified clonal. Subclonal mutations are determined by estimating the clonal frequency using a combination of the VAF, purity, coverage and copy number. Clonal mutations can be classified into early or late clonal if they occur in regions of copy number gain. This is done by first calculating the ratio of duplicated and unduplicated mutations to estimate the timing of the alteration. Mutations acquired before the gain will be present on both copies of the gained allele whereas mutations acquired after the event will not. It is only possible to time copy number losses if they are “copy number neutral” events whereby one allele is lost while the other allele is simultaneously gained. Mutations are described as early clonal or late clonal if they occur before or after the copy number change respectively. In diploid regions of the genome it is not possible to time mutations and hence they are annotated as unspecified clonal. Enrichment of early/late and clonal/subclonal driver mutations was calculated by Fisher's exact test.

9.2 Relative ordering of driver events

Given the estimates of the time at which CNAs have occurred it is possible to determine the relative ordering of driver mutations and focal CNAs. A league model approach was employed as previously described in Gerstung *et al*. (27). For each combination of driver mutations and focal CNAs, herein referred to as event A and event B, a multinomial distribution is constructed based on the likelihood of the following scenarios: event A happens before event B, event B happens before event A, or the ordering is unknown. Care must be taken when comparing mutational timings across chromosomes as timings are relative to the specific CNA on the given chromosome. To illustrate this, if a patient has a gain on chromosome 1 at 0.8 “mutational time” and a gain on chromosome 2 at 0.3 “mutational time”, then an early mutation on chromosome 2 is more likely to have occurred before an early mutation on chromosome 1. This behaviour is considered when generating the multinomial distributions. For each pair of events the multinomial distribution is sampled and points are awarded: 2 points for the event drawn occurring earlier, 0 points for the event drawn occurring

later, and 1 point for each event if unknown ordering is drawn. The event ordering is determined by the final league table after all events have been drawn against each other. The league model is repeated 1000 times and in each iteration the cohort is restricted to 75% of the sample size at random to account for anomalous samples. The final ordering distribution is aggregated across all results. An odds ratio (OR) and 95% confidence intervals are calculated empirically for each driver event to determine if they are early or late. The OR and standard error of Log(OR) is calculated as:

$$OR = \frac{N_t}{N_b};\ SE = \sqrt{\frac{1}{N_t} + \frac{1}{N_b}} \qquad (16)$$

where N_t and N_b are the number of simulations where the given event finished in the top or bottom half of the league respectively.

10. Mitochondria Profiling

10.1 Somatic variant calling of the mtDNA genome

Somatic mitochondrial SNVs and indels were called using Mutect2 (RRID: SCR_001876, v4.1.0.0, GATK v4.5.0.0; ref 81) with default settings applied. Somatic mitochondrial variants were excluded if they exhibited at least one of the following:

- Low mapping quality score (<20).
- Low base quality score (<20).
- An alternative allele frequency <1%.
- Missing alternative reads in any stand direction.
- Were within hypermutated regions; 302-316, 514-525 or 3106-3109.

10.2. Mitochondrial copy number calling

Autosomal and mitochondrial genome coverage was computed using fastMitoCalc (RRID: SCR_027724, ref 82). Mitochondrial DNA copy number was estimated as per Yuan *et al.* (83) as a function of estimated sample purity (ρ), tumour ploidy (θ) and mean coverage depth:

$$\frac{mtDNA\ mean\ coverage}{Autosomal\ DNA\ mean\ coverage}(\rho\theta + 2(1-\rho)) \qquad (17)$$

10.3. Mitochondria driver discovery

Mutational selection of mitochondrial protein-coding genes was evaluated by estimating dN/dS using the dNdScv R-package (RRID: SCR_017093; ref 42) with default parameters and isolating the mitochondrial genome as the reference genome (mtDNA genome have higher mutation rates compared to the nuclear genome). Estimation of the global mitochondrial dN/dS value excluded *MT-ND6* due to replication bias.

11. IMMUNE PROFILING

11.1. Human Leukocyte Antigen (HLA) Typing

HLA-typing of genomes was completed using POLYmorphic loci reSOLVER (POLYSOLVER, RRID: SCR_022278; ref 84), predicting the six alleles of HLA-A, HLA-B and HLA-C for each patient.

11.2 Neoantigen Prediction

Neoantigens were predicted using personalized Variant Antigens by Cancer Sequencing (RRID: SCR_025435, pVAC-Seq; ref 85), which utilises eight independent algorithms (NetMHC, NetMHCpan, MHCflurry, SMM, NetMHCcons, SMMPMBEC, MHCnuggetsI, PickPocket) to predict the binding affinities of epitopes to MHC. The mean binding score was calculated and used as the neoantigen binding strength.

pVAC-Seq was run independently on each tumour sample using patient specific HLA alleles. The binding scores of epitopes, arising due to non-synonymous mutations, are predicted for all patients. pVAC-Seq considers the transcriptional context of mutations to determine the possible wild-type and mutant-type (8-10-mer) peptides arising from each non-synonymous mutation. A given peptide is annotated as a potential neoantigen if the peptide meets the following conditions:

- Has a binding affinity ≤500nM.
- Is present in a canonical transcript.
- Is novel with respect to the human proteome.

11.3 Immune Escape

Genetically predicted immune escape was determined by considering three mechanisms, specifically: (i) a non-synonymous mutation in any of HLA-A, HLA-B or HLA-C; (ii) loss of heterozygosity (LOH) in any of the

three HLA-I genes or (iii) an inactivating mutation in any of 22 antigen presenting genes (APGs). Tumours were annotated with positive immune escape status on the basis of any one of (i)-(iii).

Mutations in HLA-A, HLA-B and HLA-C were found using POLYSOLVER, using MuTect to detect nonsynonymous SNVs and Strelka for insertions and deletions in HLA-aligned reads. LOH in HLA-I genes was predicted using Loss of Heterozygosity in Human Leukocyte Antigen (LOHHLA, RRID: SCR_023690; ref 86) using the following options: mapping and fishing steps turned on; number of mismatch sites between any two allele pairs >10; minimum coverage = 10. By default, LOHHLA considers the HLA region based on hg19 and so the source code was edited to utilise the hg38 HLA-region chr6:28510120-33480577.

LOH was defined on the basis of Cornish et. al. (87), i.e,

- Presence of allelic imbalance, defined by $P < 0.01$.
- The copy number of the lost allele <0.5 with a confidence interval <0.7.
- The copy number of the kept allele was >0.7.
- The number of mismatched sites between alleles was >10.

We curated a list of 22 APGs documented to regulate antigen presenting machinery: the IFN-γ pathway, the PF-L1 receptor, the CD58 receptor, and epigenetic escape via *SETDB1* (*APLNR, B2M, CANX, CALR, CD274, CD58, CIITA, ERAP1, ERAP2, IRF2, IFNGR1, IFGNR2, JAK1, JAK2, NLRC5, PDIA3, RFX5, SETDB1, STAT1, TAPBP, TAP1, TAP2*) (88,89). Excluding *CD274* and *SETDB1*, a gene was labelled as inactivated if any one of the following three conditions were met:

- Monoalleic or bialleic clonal loss-of-function mutation annotated with any of the VEP calculated consequences: ‘frameshift variant’, ‘stop gained’, ‘stop lost’, ‘splice acceptor variant’, ‘splice donor variant’, ‘splice region variant’ or ‘start lost’.
- Biallelic clonal non-synonymous mutation, or a monoalleic clonal non-synonymous mutation plus loss of heterozygosity, annotated with any of the following VEP calculated consequences: ‘transcript ablation’, ‘transcript amplification’, ‘inframe insertion’, ‘inframe deletion’, ‘missense variant’ or ‘protein altering variant’.
- Homozygous deletion.

As increased *CD274* and *SETDB1* expression are predicted to suppress the adaptive immune system, we considered amplification of these genes (total copy number > 3) as a mechanism of genetically immune escape.

12. CORRELATING CLINICOPATHOLOGICAL AND MUTATIONAL VARIABLES

12.1 CORRELATIONS WITH MUTATIONAL ATTRIBUTES

Correlations between clinical features (sex, stratified age (>45 years), stage and grade) with genetic/molecular variables were assessed via Fisher's exact tests, and otherwise univariate linear regression or negative binomial regression was applied for continuous and count features respectively (**Supplementary Table S16-20**). Tumour stage was summarised as a function of TNM staging using the following criteria for renal cell carcinoma:

Stage 1: T1 stage.

Stage 2: T2 stage.

Stage 3: T3 stage or N1 stage.

Stage 4: T4 stage or M1 stage.

Correlations between genetic/molecular features applied multivariate negative binomial regression, linear regression and logistic regression (adjusting for patient sex, age of sampling and tumour stage; **Supplementary Table S16-20**). Some associations are not reported due to a lack of events in binary variables. A two-sided *P* <0.05 being considered statistically significant

Supplementary Method References

1. Turnbull C. Introducing whole-genome sequencing into routine cancer care: the Genomics England 100 000 Genomes Project. Ann Oncol. 2018;29:784–7.

2. Turnbull C, Scott RH, Thomas E, Jones L, Murugaesu N, Pretty FB, et al. The 100 000 Genomes Project: bringing whole genome sequencing to the NHS. BMJ. 2018;361:k1687.

3. Moch H, Amin MB, Berney DM, Compérat EM, Gill AJ, Hartmann A, et al. The 2022 World Health Organization Classification of Tumours of the Urinary System and Male Genital Organs-Part A: Renal, Penile, and Testicular Tumours. Eur Urol. 2022;82:458–68.

4. The National Genomic Research Library v5.1, Genomics England. 2020; Available from: http://dx.doi.org/10.6084/m9.figshare.4530893.v7

5. Raczy C, Petrovski R, Saunders CT, Chorny I, Kruglyak S, Margulies EH, et al. Isaac: ultra-fast whole-genome secondary analysis on Illumina sequencing platforms. Bioinformatics. 2013;29:2041–3.

6. Saunders CT, Wong WSW, Swamy S, Becq J, Murray LJ, Cheetham RK. Strelka: accurate somatic small-variant calling from sequenced tumor–normal sample pairs. Bioinformatics. 2012;28:1811–7.

7. Karczewski KJ, Francioli LC, Tiao G, Cummings BB, Alföldi J, Wang Q, et al. The mutational constraint spectrum quantified from variation in 141,456 humans. Nature. 2020;581:434–43.

8. Tate JG, Bamford S, Jubb HC, Sondka Z, Beare DM, Bindal N, et al. COSMIC: the Catalogue Of Somatic Mutations In Cancer. Nucleic Acids Res. 2018;47:D941–7.

9. Benson G. Tandem repeats finder: a program to analyze DNA sequences. Nucleic Acids Res. 1999;27:573–80.

10. Jamal-Hanjani M, Hackshaw A, Ngai Y, Shaw J, Dive C, Quezada S, et al. Tracking genomic cancer evolution for precision medicine: the lung TRACERx study. PLoS Biol. 2014;12:e1001906.

11. Cornish AJ, Chubb D, Frangou A, Hoang PH, Kaiser M, Wedge DC, et al. Reference bias in the Illumina Isaac aligner. Bioinformatics. 2020;36:4671–2.

12. Salipante SJ, Scroggins SM, Hampel HL, Turner EH, Pritchard CC. Microsatellite Instability Detection by Next Generation Sequencing. Clin Chem. 2014;60:1192–9.

13. Beier S, Thiel T, Münch T, Scholz U, Mascher M. MISA-web: a web server for microsatellite prediction. Bioinformatics. 2017;33:2583–5.

14. Nik-Zainal S, Van Loo P, Wedge DC, Alexandrov, B. L, Greenman CD, et al. The Life History of 21 Breast Cancers. Cell. 2012;149:994–1007.

15. Delaneau O, Marchini J, Zagury J-F. A linear complexity phasing method for thousands of genomes. Nat Methods. 2011;9:179–81.

16. Nilsen G, Liestøl K, Van Loo P, Moen Vollan HK, Eide MB, Rueda OM, et al. Copynumber: Efficient algorithms for single- and multi-track copy number segmentation. BMC Genomics. 2012;13:591.

17. Van Loo P, Nordgard SH, Lingjærde OC, Russnes, Hege G., Rye, Inga H., Sun W, Weigman VJ, et al. Allele-specific copy number analysis of tumors. PNAS. 2010;107:16910–5.

18. Dentro SC, Wedge DC, Van Loo P. Principles of Reconstructing the Subclonal Architecture of Cancers. Cold Spring Harb Perspect Med. 2017;7:a026625.

19. Weber CM, Ramachandran S, Henikoff S. Nucleosomes are context-specific, H2A.Z-modulated barriers to RNA polymerase. Mol Cell. 2014;53:819–30.

20. Rausch T, Zichner T, Schlattl A, Stütz AM, Benes V, Korbel JO. DELLY: structural variant discovery by integrated paired-end and split-read analysis. Bioinformatics. 2012;28:i333–9.

21. Layer RM, Chiang C, Quinlan AR, Hall IM. LUMPY: a probabilistic framework for structural variant discovery. Genome Biol. 2014;15:R84.

22. Chen X, Schulz-Trieglaff O, Shaw R, Barnes B, Schlesinger F, Källberg M, et al. Manta: rapid detection of structural variants and indels for germline and cancer sequencing applications. Bioinformatics. 2016;32:1220–2.

23. Li Y, Roberts ND, Wala JA, Shapira O, Schumacher SE, Kumar K, et al. Patterns of somatic structural variation in human cancer genomes. Nature. 2020;578:112–21.

24. Rodriguez-Martin B, Alvarez EG, Baez-Ortega A, Zamora J, Supek F, Demeulemeester J, et al. Pan-cancer analysis of whole genomes identifies driver rearrangements promoted by LINE-1 retrotransposition. Nat Genet. 2020;52:306–19.

25. Chu C, Borges-Monroy R, Viswanadham VV, Lee S, Li H, Lee EA, et al. Comprehensive identification of transposable element insertions using multiple sequencing technologies. Nat Commun. 2021;12:3836.

26. Tubio JMC, Li Y, Ju YS, Martincorena I, Cooke SL, Tojo M, et al. Mobile DNA in cancer. Extensive transduction of nonrepetitive DNA mediated by L1 retrotransposition in cancer genomes. Science. 2014;345:1251343.

27. Gerstung M, Jolly C, Leshchiner I, Dentro SC, Gonzalez S, Rosebrock D, et al. The evolutionary history of 2,658 cancers. Nature. 2020;578:122–8.

28. Farmery JHR, Smith ML, NIHR BioResource - Rare Diseases, Lynch AG. Telomerecat: A ploidy-agnostic method for estimating telomere length from whole genome sequencing data. Sci Rep. 2018;8:1300.

29. Deshpande V, Luebeck J, Nguyen N-PD, Bakhtiari M, Turner KM, Schwab R, et al. Exploring the landscape of focal amplifications in cancer using AmpliconArchitect. Nat Commun. 2019;10:392.

30. Luebeck J, Ng AWT, Galipeau PC, Li X, Sanchez CA, Katz-Summercorn AC, et al. Extrachromosomal DNA in the cancerous transformation of Barrett's oesophagus. Nature. 2023;616:798–805.

31. Avulova S, Cheville JC, Lohse CM, Potretzke TA, Tsivian M, Thompson RH, et al. Grading Chromophobe Renal Cell Carcinoma: Evidence for a Four-tiered Classification Incorporating Coagulative Tumor Necrosis. European Urology. 2021;79:225–31.

32. Delahunt B, Sika-Paotonu D, Bethwaite PB, McCredie MRE, Martignoni G, Eble JN, et al. Fuhrman grading is not appropriate for chromophobe renal cell carcinoma. Am J Surg Pathol. 2007;31:957–60.

33. Dentro SC, Leshchiner I, Haase K, Tarabichi M, Wintersinger J, Deshwar AG, et al. Characterizing genetic intra-tumor heterogeneity across 2,658 human cancer genomes. Cell. 2021;184:2239–54.e39.

34. The ICGC/TCGA Pan-Cancer Analysis of Whole Genomes Consortium. Pan-cancer analysis of whole genomes. Nature. 2020;578:82–93.

35. McLaren W, Gil L, Hunt SE, Riat HS, Ritchie GRS, Thormann A, et al. The Ensembl Variant Effect Predictor. Genome Biol. 2016;17:122.

36. Kircher M, Witten DM, Jain P, O'Roak BJ, Cooper GM, Shendure J. A general framework for estimating the relative pathogenicity of human genetic variants. Nat Genet. 2014;46:310–5.

37. Rentzsch P, Witten D, Cooper GM, Shendure J, Kircher M. CADD: predicting the deleteriousness of variants

throughout the human genome. Nucleic Acids Res. 2019;47:D886–94.

38. Rentzsch P, Schubach M, Shendure J, Kircher M. CADD-Splice-improving genome-wide variant effect prediction using deep learning-derived splice scores. Genome Med. 2021;13:31.

39. Zhang X, Wakeling M, Ware J, Whiffin N. Annotating high-impact 5'untranslated region variants with the UTRannotator. Bioinformatics. 2021;37:1171–3.

40. Martínez-Jiménez F, Muiños F, Sentís I, Deu-Pons J, Reyes-Salazar I, Arnedo-Pac C, et al. A compendium of mutational cancer driver genes. Nat Rev Cancer. 2020;20:555–72.

41. Priestley P, Baber J, Lolkema MP, Steeghs N, de Bruijn E, Shale C, et al. Pan-cancer whole-genome analyses of metastatic solid tumours. Nature. 2019;575:210–6.

42. Martincorena I, Raine KM, Gerstung M, Dawson KJ, Haase K, Van Loo P, et al. Universal Patterns of Selection in Cancer and Somatic Tissues. Cell. 2017;1731:1029–41.

43. Mularoni L, Sabarinathan R, Deu-Pons J, Gonzalez-Perez A, López-Bigas N. OncodriveFML: a general framework to identify coding and non-coding regions with cancer driver mutations. Genome Biol. 2016;17:128.

44. Arnedo-Pac C, Mularoni L, Muiños F, Gonzalez-Perez A, Lopez-Bigas N. OncodriveCLUSTL: a sequence-based clustering method to identify cancer drivers. Bioinformatics. 2019;35:5396.

45. Weghorn D, Sunyaev S. Bayesian inference of negative and positive selection in human cancers. Nat Genet. 2017;49:1785–8.

46. Dietlein F, Weghorn D, Taylor-Weiner A, Richters A, Reardon B, Liu D, et al. Identification of cancer driver genes based on nucleotide context. Nat Genet. 2020;52:208–18.

47. Tokheim C, Bhattacharya R, Niknafs N, Gygax DM, Kim R, Ryan M, et al. Exome-Scale Discovery of Hotspot Mutation Regions in Human Cancer Using 3D Protein Structure. Cancer Res. 2016;76:3719–31.

48. Burley SK, Bhikadiya C, Bi C, Bittrich S, Chen L, Crichlow GV, et al. RCSB Protein Data Bank: powerful new tools for exploring 3D structures of biological macromolecules for basic and applied research and education in fundamental biology, biomedicine, biotechnology, bioengineering and energy sciences. Nucleic Acids Res. 2021;49:D437–51.

49. Porta-Pardo E, Godzik A. e-Driver: a novel method to identify protein regions driving cancer. Bioinformatics. 2014;30:3109–14.

50. Sondka Z, Bamford S, Cole CG, Ward SA, Dunham I, Forbes SA. The COSMIC Cancer Gene Census: describing genetic dysfunction across all human cancers. Nat Rev Cancer. 2018;18:696–705.

51. Gudmundsson S, Singer-Berk M, Watts NA, Phu W, Goodrich JK, Solomonson M, et al. Variant interpretation using population databases: Lessons from gnomAD. Hum Mutat. 2021; 43:1012-1030

52. Lever J, Zhao EY, Grewal J, Jones MR, Jones SJM. CancerMine: a literature-mined resource for drivers, oncogenes and tumor suppressors in cancer. Nat Methods. 2019;16:505–7.

53. Bailey MH, Tokheim C, Porta-Pardo E, Sengupta S, Bertrand D, Weerasinghe A, et al. Comprehensive Characterization of Cancer Driver Genes and Mutations. Cell. 2018;173:371–85.

54. Chakravarty D, Gao J, Phillips SM, Kundra R, Zhang H, Wang J, et al. OncoKB: A Precision Oncology Knowledge Base. JCO Precis Oncol. 2017;2017:1–16.

55. Paczkowska M, Barenboim J, Sintupisut N, Fox NS, Zhu H, Abd-Rabbo D, et al. Integrative pathway enrichment analysis of multivariate omics data. Nat Commun. 2020;11:735.

56. Subramanian A, Tamayo P, Mootha VK, Mukherjee S, Ebert BL, Gillette MA, et al. Gene set enrichment analysis: a knowledge-based approach for interpreting genome-wide expression profiles. Proc Natl Acad Sci U S A. 2005;102:15545–50.

57. Liberzon A, Birger C, Thorvaldsdóttir H, Ghandi M, Mesirov JP, Tamayo P. The Molecular Signatures Database (MSigDB) hallmark gene set collection. Cell Syst. 2015;1:417–25.

58. Martin FJ, Amode MR, Aneja A, Austine-Orimoloye O, Azov AG, Barnes I, et al. Ensembl 2023. Nucleic Acids Res. 2023;51:D933–41.

59. Neph S, Kuehn MS, Reynolds AP, Haugen E, Thurman RE, Johnson AK, et al. BEDOPS: high-performance genomic feature operations. Bioinformatics. 2012;28:1919–20.

60. Zhu H, Uusküla-Reimand L, Isaev K, Wadi L, Alizada A, Shuai S, et al. Candidate Cancer Driver Mutations in Distal Regulatory Elements and Long-Range Chromatin Interaction Networks. Mol Cell. 2020;77:1307–21.e10.

61. Mermel CH, Schumacher SE, Hill B, Meyerson ML, Beroukhim R, Getz G. GISTIC2.0 facilitates sensitive and confident localization of the targets of focal somatic copy-number alteration in human cancers. Genome Biology. 2011;12:R41.

62. Dale RK, Pedersen BS, Quinlan AR. Pybedtools: a flexible Python library for manipulating genomic datasets and annotations. Bioinformatics. 2011;27:3423–4.

63. Quinlan AR, Hall IM. BEDTools: a flexible suite of utilities for comparing genomic features. Bioinformatics. 2010;26:841–2.

64. Zack TI, Schumacher SE, Carter SL, Cherniack AD, Saksena G, Tabak B, et al. Pan-cancer patterns of somatic copy number alteration. Nat Genet. 2013;45:454–65.

65. Cortés-Ciriano I, Lee JJ-K, Xi R, Jain D, Jung YL, Yang L, et al. Comprehensive analysis of chromothripsis in 2,658 human cancers using whole-genome sequencing. Nat Genet. 2020;52:331–41.

66. Korbel JO, Campbell PJ. Criteria for inference of chromothripsis in cancer genomes. Cell. 2013;152:1226–36.

67. Glodzik D, Morganella S, Davies H, Simpson PT, Li Y, Zou X, et al. A somatic-mutational process recurrently duplicates germline susceptibility loci and tissue-specific super-enhancers in breast cancers. Nat Genet. 2017;49:341–8.

68. Haeussler M, Zweig AS, Tyner C, Speir ML, Rosenbloom KR, Raney BJ, et al. The UCSC Genome Browser database: 2019 update. Nucleic Acids Res. 2019;47:D853–8.

69. She X, Jiang Z, Clark RA, Liu G, Cheng Z, Tuzun E, et al. Shotgun sequence assembly and recent segmental duplications within the human genome. Nature. 2004;431:927–30.

70. Weddington N, Stuy A, Hiratani I, Ryba T, Yokochi T, Gilbert DM. ReplicationDomain: a visualization tool and comparative database for genome-wide replication timing data. BMC Bioinformatics. 2008;9:530.

71. The ENCODE Project Consortium. An integrated encyclopedia of DNA elements in the human genome. Nature. 2012;489:57–74.

72. Luo Y, Hitz BC, Gabdank I, Hilton JA, Kagda MS, Lam B, et al. New developments on the Encyclopedia of DNA Elements (ENCODE) data portal. Nucleic Acids Res. 2020;48:D882–9.

73. Bignell GJ, Greenman CD, Davies H, Butler AP, Edkins S, Andrews JM, et al. Signatures of mutation and selection in the cancer genome. Nature. 463:893–8.

74. Barlow JH, Faryabi RB, Callén E, Wong N, Malhowski A, Chen HT, et al. Identification of early replicating fragile

sites that contribute to genome instability. Cell. 2013;152:620–32.

75. Beroukhim R, Mermel CH, Porter D, Wei G, Raychaudhuri S, Donovan J, et al. The landscape of somatic copy-number alteration across human cancers. Nature. 2010;463:899–905.

76. Le Tallec B, Millot GA, Blin ME, Brison O, Dutrillaux B, Debatisse M. Common fragile site profiling in epithelial and erythroid cells reveals that most recurrent cancer deletions lie in fragile sites hosting large genes. Cell Rep. 2013;4:420–8.

77. Islam SMA, Díaz-Gay M, Wu Y, Barnes M, Vangara R, Bergstrom EN, et al. Uncovering novel mutational signatures by de novo extraction with SigProfilerExtractor. Cell Genom. 2022;2:100179.

78. Everall A, Tapinos A, Hawari A, Cornish A, Sud A, Chubb D, et al. Comprehensive repertoire of the chromosomal alteration and mutational signatures across 16 cancer types from 10,983 cancer patients. medrxiv. 2023;2023.06.07.23290970.

79. Davies H, Glodzik D, Morganella S, Yates LR, Staaf J, Zou X, et al. HRDetect is a predictor of BRCA1 and BRCA2 deficiency based on mutational signatures. Nat Med. 2017;23:517–25.

80. Nik-Zainal S, Davies H, Staaf J, Ramakrishna M, Glodzik D, Zou X, et al. Landscape of somatic mutations in 560 breast cancer whole-genome sequences. Nature. 2016;534:47–54.

81. Benjamin D, Sato T, Cibulskis K, Getz G, Stewart C, Lichtenstein L. Calling Somatic SNVs and Indels with Mutect2 [Internet]. bioRxiv. 2019 [cited 2025 Mar 28]. page 861054. Available from: https://www.biorxiv.org/content/10.1101/861054v1.abstract

82. Qian Y, Butler TJ, Opsahl-Ong K, Giroux NS, Sidore C, Nagaraja R, et al. fastMitoCalc: an ultra-fast program to estimate mitochondrial DNA copy number from whole-genome sequences. Bioinformatics. 2017;33:1399–401.

83. Yuan Y, Ju YS, Kim Y, Li J, Wang Y, Yoon CJ, et al. Comprehensive molecular characterization of mitochondrial genomes in human cancers. Nature Genetics. 2020;52:342–52.

84. Shukla SA, Rooney MS, Rajasagi M, Tiao Grace Dixon Philip, Lawrence MS, Stevens J, et al. Comprehensive analysis of cancer-associated somatic mutations in class I HLA genes. Nat Biotechnol. 2015;33:1152–8.

85. Hundal J, Carreno BM, Petti AA, Linette GP, Griffith OL, Mardis ER, et al. pVAC-Seq: A genome-guided in silico approach to identifying tumor neoantigens. Genome Med. 2016;8:11.

86. McGranahan N, Rosenthal R, Hiley CT, Rowan AJ, Watkins TBK, Wilson GA, et al. Allele-Specific HLA Loss and Immune Escape in Lung Cancer Evolution. Cell. 2017;171:1259–71.e11.

87. Cornish AJ, Gruber AJ, Kinnersley B, Chubb D, Frangou A, Caravagna G, et al. The genomic landscape of 2,023 colorectal cancers. Nature. 2024;633:127–36.

88. Kelly A, Trowsdale J. Genetics of antigen processing and presentation. Immunogenetics. 2023;71:161–70.

89. Martínez-Jiménez F, Priestley P, Shale C, Baber J, Rozemuller E, Cuppen E. Genetic immune escape landscape in primary and metastatic cancer. Nat Genet. 2023;55:820–31.
